# Altered Effective Connectivity of the Numerical Brain in Children With Developmental Dyscalculia

**DOI:** 10.1002/jnr.70066

**Published:** 2025-07-21

**Authors:** Simone Schwizer Ashkenazi, Ursina McCaskey, Ruth O’Gorman Tuura, Karin Kucian

**Affiliations:** ^1^ Center for MR‐Research, University Children’s Hospital Zurich Zurich Switzerland; ^2^ Neuropsychology, Department of Psychology University of Zurich Zurich Switzerland; ^3^ Children’s Research Center, University Children’s Hospital Zurich Zurich Switzerland; ^4^ Zurich Center for Integrative Human Physiology, University of Zurich Zurich Switzerland; ^5^ Neuroscience Center Zurich, University of Zurich and Swiss Federal Institute of Technology Zurich Zurich Switzerland

**Keywords:** children, developmental dyscalculia, effective connectivity, intraparietal sulcus, MRI, numerical abilities, pre‐supplementary motor area

## Abstract

Numerical‐order ability, a strong predictor of arithmetic, is often impaired in children with developmental dyscalculia (DD). While previous research has shown altered brain responses in number‐processing regions in DD compared to typically developing children (TD), little is known about how these regions interact during number processing. This exploratory study examined the effective connectivity between six regions in the right parietal, frontal, and insular cortex as well as the vermis, using dynamic causal modeling (DCM). We investigated how number‐order and number‐identification tasks modulate connectivity within this network and the group differences related to DD. The number‐order task led primarily to increased excitatory connectivity from the pre‐supplementary motor area (preSMA) to all other regions, indicating an orchestrating role of the preSMA. DD, who exhibited deficits in number‐order performance, demonstrated aberrant modulation of incoming connectivity to the ventral premotor cortex (vPMC) from the anterior intraparietal sulcus (aIPS), the preSMA, and the dorsal anterior insula (d‐aINS). In TD, number‐identification led to inhibitory modulation from the vPMC to the aIPS and the vermis. While behavioral performance in number‐identification was unimpaired in DD, they showed increased excitatory connectivity from dorsal and ventral PMC to the d‐aINS and from vPMC to the aIPS. Our results imply that, for both impaired and unimpaired number‐related behavioral performance, neuronal number processing differs between DD and TD. This conclusion is further supported by the high predictive validity of the modulating connectivity group‐effect parameters. We suggest the underlying explanation for this pattern may be related to decreased acuity of neuronal number representation in DD.

## Graphical abstract

We examined effective brain connectivity in children with and without developmental dyscalculia (DD) during number order and identification tasks. Results highlight a central role of the pre‐supplementary motor area in number ordering. Children with DD showed altered connectivity in both tasks, despite intact behavioral performance in number identification.

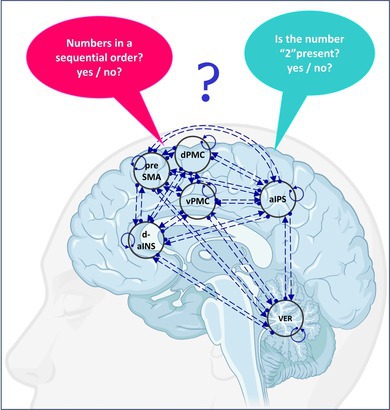

**Summary**

- The ability to identify whether numbers appear in sequential order, crucial for arithmetic, are often impaired in children with developmental dyscalculia (DD). Our study examined how brain regions communicate during number tasks in both DD and typically developing children (TD).
- We found that a brain area associated with cognitive control (pre‐supplementary motor area) orchestrates all other involved brain regions to coordinate number order processing.
- Children with DD show different patterns in the communication between certain brain areas, which likely are related to their arithmetic difficulties.
- Understanding these brain differences can help to develop better educational strategies and interventions for children with DD.

**Abbreviations**
aIPS: anterior intraparietal sulcus
A‐matrix: common effective connectivity across conditions
ANS: approximate number system
A‐parameter: endogenous parameter across conditions
BMA: Bayesian model averaging
B‐matrix: modulation on common effective connectivity
B‐parameter: modulatory parameter
d‐aINS: dorsal portion of the anterior insula
DCM: dynamic causal modeling
DD: developmental dyscalculia
DMN: default mode network
dPMC: dorsal premotor cortex
FPN: fronto‐parietal network
OTS: object tracking system
PEB: parametric empirical Bayes
PMC: premotor cortex
preSMA: pre‐supplementary motor area
SPC: superior parietal cortex
STS: scan to scan
TD: typically developed
VER‐VI: vermis lobule VI
vPMC: ventral premotor cortex
WISC: Wechsler Intelligence Scale for Children
ZAREKI‐R: Neuropsychological Test Battery for Number Processing and Calculation in Children—Revised

## 1 Introduction

Developmental dyscalculia (DD) is a math learning disorder affecting an individual's ability to understand and manipulate numbers or quantities, and mathematical concepts (Castaldi et al. 2020). These deficits impact daily and professional life (Castaldi et al. 2020; Vigna et al. 2022) and are reflected in aberrant brain function and structure compared to typically developing (TD) individuals (Tablante et al. 2023). Despite interventions showing improvements in numerical abilities and optimizing functional brain reorganization (Kucian et al. 2011; Michels et al. 2018), longitudinal studies demonstrate persistent impairments in math skills and altered brain measures in individuals with DD (McCaskey et al. 2018, 2020; Nelson and Powell 2018).

The cognitive mechanisms underlying DD continue to be debated. Children with DD show particular deficits in symbolic number processing (Schwenk et al. 2017) including numerical‐order ability (Devlin et al. 2022; Morsanyi et al. 2018) and arithmetic (Peters and De Smedt 2018) and to a lesser degree in non‐symbolic number processing (Olsson et al. 2016; Schwenk et al. 2017). These deficits are likely the result of multiple contributing factors, including domain‐specific impairments in the approximate number system (ANS) and object tracking system (OTS), difficulties in accessing numerosity through symbolic numbers, and domain‐general weaknesses in working memory and executive functions (Vogel and De Smedt 2021). The extent and combination of these factors may vary across individuals.

Numerical abilities have been in particular associated with a fronto‐parietal brain network (Moeller et al. 2015) and metadata analysis revealed that the intraparietal sulcus is most consistently involved in number processing (Escobar‐Magariño et al. 2022). A series of recent studies have identified neuronal populations in the parietal and frontal lobes that are tuned for numerosity across various tasks and modalities (Harvey et al. 2013; Hofstetter et al. 2021) including naturalistic images containing real‐world objects (Hofstetter and Dumoulin 2022) thereby supporting the ecological validity of numerosity tuning mechanisms. Additional regions implicated in numerical processing include the insula, lateral and medial temporal areas, and the cerebellum (Arsalidou et al. 2018; Kucian and von Aster 2015; McCaskey et al. 2018; Peters and De Smedt 2018). Arsalidou et al. (2018) conducted a meta‐analysis comparing TD Children’s brain activation during number and calculation tasks. For basic number tasks that did not require mathematical operations—comprising both symbolic and non‐symbolic stimuli—activation demonstrated strong right hemisphere dominance, including the inferior parietal lobe, postcentral gyrus, claustrum, and insula. When tasks incorporated calculation alongside number presentation, the right hemispheric dominance persisted with additional activity in the right cingulate and precuneus, while also recruiting left claustrum and bilateral structures including medial frontal gyrus near the frontal eye fields, pre‐supplementary motor area, and insula (Arsalidou et al. 2018). These findings highlight not only the recruitment of broader neural networks when numerical processing in TD children extends beyond basic perception to more complex operations but also the consistent right‐lateralized pattern of activation across both number processing and calculation tasks in children.

Individuals with DD show widespread neural differences compared to TD counterparts. A recent meta‐analysis by Tablante et al. (2023) identified a nuanced pattern of alterations: decreased activation in temporal regions and anterior IPS, but increased activation in posterior IPS, frontal regions, and insula. Notably, the right anterior IPS emerged as the most consistently altered region in DD. These findings refine earlier research that primarily reported decreased parietal activation in DD (Berteletti et al. 2014; Kucian et al. 2011; Mussolin et al. 2010), though some studies noted increased parietal activations (Kaufmann et al. 2009; Rosenberg‐Lee et al. 2015). Furthermore, De Smedt et al. (2019) highlighted a consistent finding across brain activation studies: TD children show difficulty‐related modulation of the fronto‐parietal network during problem‐solving, whereas children with DD fail to demonstrate such modulation. De Smedt and colleagues suggested this neural pattern might reflect that children with DD continue to rely on immature procedural strategies for both easy and complex problems, while TD children shift to fact retrieval strategies for simpler problems, as observed in behavioral studies (Geary 2011). The failure in DD to adaptively engage neural resources likely stems from atypical neurodevelopment of these critical regions and their connections, potentially underlying the persistent arithmetic difficulties associated with DD. Indeed, beyond functional differences, structural alterations have been observed in fronto‐parietal brain regions (Rotzer et al. 2008; McCaskey et al. 2020) and connecting white matter tracts (Kucian et al. 2014; Rykhlevskaia et al. 2009).

While our understanding of localized brain activity during numerical processing has advanced, less is known about how brain regions interact as networks. Connectivity studies in TD children have shown that resting state intrinsic functional connectivity of the hippocampus with basal ganglia and dorso‐ and ventrolateral prefrontal cortex predicts gains in arithmetic (Supekar et al. 2013), while functional connectivity between inferior frontal region and the intraparietal sulcus correlates with basic numerical skills (i.e., matching symbolic and non‐symbolic quantity) and the score of a standardized math test (Emerson and Cantlon 2012). Research in very young TD children has shown task‐based functional connectivity using psychophysiological interaction analysis from a seed region in the right superior parietal cortex (SPC) to the left supramarginal gyrus and to the right premotor cortex (PMC) during symbolic versus non‐symbolic magnitude comparison, with the strength of the later connectivity (SPC to PMC) predicting symbolic mathematical skills (Park et al. 2014).

In children with DD, studies investigating seed‐based functional connectivity point to an aberrant hyperconnectivity between the IPS (seed) and several regions mainly in the frontal cortex in resting state (Jolles et al. 2016) as well as task‐based functional connectivity during the solving of arithmetic additions and subtractions (Rosenberg‐Lee et al. 2015) or ordinal processing of symbolic numbers (Michels et al. 2018).

The detection of hyperconnectivity in DD at first glance appears to contradict findings of reduced white matter structure connecting parietal and frontal regions. However, structural brain impairment can lead to both increases or decreases in functional interactions of brain regions (Horwitz et al. 2013). Hyperconnectivity has been observed in other disorders and may represent either aberrant functional networks or compensatory mechanisms (Iraji et al. 2016; Krishnadas et al. 2014; Pagani et al. 2021).

A critical question that remains largely unexplored is how brain regions involved in numerical processing causally interact in TD children and whether these interactions differ in children with DD. Examining effective connectivity can shed light on these mechanisms by uncovering how information flows within the network during number processing. Group differences in effective connectivity may point to fundamental alterations in the organization networks involved in number processing in DD: either reflecting potential impairments in the hierarchical coordination of brain regions or compensatory strategies that emerge in response to underlying network inefficiencies. To our knowledge, no study has comprehensively investigated effective connectivity by exploring causal influences among multiple regions of a numerical network in children with DD compared to TD children.

This exploratory study aims to address this gap by providing initial insights into typical and atypical effective connectivity within a brain network associated with symbolic number ordering, given its significance for arithmetic, mathematical competence, and DD (Lyons and Ansari 2015; Morsanyi et al. 2018, 2020; Sommerauer et al. 2020). The network included the following regions: the right anterior intraparietal sulcus (aIPS), right dorsal and ventral premotor cortex (dPMC, vPMC), right pre‐supplementary motor area (preSMA), dorsal portion of the anterior insula (d‐aINS), and vermis lobule VI (VER‐VI). All these regions have been previously implicated in numerical cognition. The right aIPS is consistently activated in number processing tasks in children (Arsalidou et al. 2018) and shows altered activation in DD (Tablante et al. 2023). Premotor regions are associated with sequence processing and finger‐based representations of numerosity (Andres and Pesenti 2015; Krinzinger et al. 2011; Tschentscher et al. 2012). The preSMA is involved in cognitive control and mediating sequence processing across various domains, including numerical cognition (Cona and Semenza 2017). The right d‐aINS is known for its role in cognitive control and salience processing. It acts as a filter and integration hub, identifying the most relevant among competing internal and external stimuli (Molnar‐Szakacs and Uddin 2022; Seeley 2019; Uddin et al. 2017). Furthermore, the insula has been associated with mental arithmetic procedural processes in a meta‐analysis (Sokolowski et al. 2023). Finally, the cerebellar vermis has been implicated in number processing in adults in a study contrasting calculation with number identification (Wu et al. 2009), and in youth, as shown by a conjunction fMRI analysis of non‐symbolic quantity judgment, area magnitude judgment, and mental rotation tasks (McCaskey et al. 2017). This region has also been reported to be involved in cognitive flexibility (Berger et al. 2005; Ravizza and Carter 2008; Woolley et al. 2008)., Vermis lobule VI in particular, has been associated with the automatic, bottom‐up detection of salient stimuli (Habas 2022; Habas et al. 2009). Thus, our data‐driven ROI selection is grounded in and supported by the theoretical and empirical literature on numerical cognition.

We applied dynamic causal modeling (DCM) (Friston et al. 2003; Zeidman, Jafarian, Corbin, et al. 2019; Zeidman, Jafarian, Seghier, et al. 2019) and the parametric empirical Bayes (PEB) framework to analyze the interactions between six regions of interest (ROI) and their self‐inhibitions. As no prior studies have comprehensively examined effective connectivity during numerical processing in typically developing (TD) children and those with developmental dyscalculia (DD), we adopted an exploratory approach and implemented a full model by including all possible connections. We evaluated the modulatory effect of number ordering and number identification on the common effective connectivity across conditions and the group effect by DD.

We hypothesized that children with DD compared to TD would exhibit significantly poorer performance in the number order condition (Devlin et al. 2022; Morsanyi et al. 2018), but not in the number identification condition. Furthermore, we expected that DD would show in particular impairments in symbolic and non‐symbolic number comparison and arithmetic tasks, based on previous findings (Olsson et al. 2016; Peters and De Smedt 2018; Schwenk et al. 2017). As stated above, we followed an exploratory approach and formulated broad expectations rather than specific directional hypotheses regarding network connectivity. Across all participants, we anticipated that the number order task would elicit more excitatory connectivity and reduced self‐inhibition, reflecting its higher cognitive demands. In contrast, we expected the number identification task to be associated with more inhibitory connectivity and stronger self‐inhibition. Finally, we predicted that group differences related to DD would emerge during the number order task, but not during number identification. In total, the present study represents a first step toward a better understanding of the neural network dynamics underlying number processing and its relevance for mathematical development and dyscalculia.

## 2 Method

### 2.1 Study Design and Participants

To address our research questions, we analyzed data from 45 children with and without DD, combining participants from two previously published datasets (Kucian et al. 2011; McCaskey et al. 2018) with nine additional children recruited at our center whose data have not been previously published. This combined dataset included functional and structural MRI data as well as behavioral assessments. Inclusion criteria for all participants included no history of neurological or psychological disorders and an Intelligence Quotient (IQ) ≥ 85, as estimated by the Wechsler Intelligence Scale for Children, third edition (WISC‐III; Tewes et al. 1999). Five participants were excluded due to motion artifacts (see Section 2.3.2), nine participants were excluded because they did not show activation in all ROIs (see Section 2.4.2.2.1), and one additional participant was excluded due to insufficient explained variance (see Section 2.4.2.2.2). This resulted in a final sample of 30 children (15 with DD and 15 TD children) who were included in the statistical analyses. Demographic information for both groups is presented in Table 1.

**TABLE 1:** Demographic characteristics of DD and TD groups.

Variable	TD group (*n* = 15)	DD group (*n* = 15)
Age (years)	*M* = 9.7, SD = 0.9 (Range: 8.3–11.3)	*M* = 9.9, SD = 1.0 (Range: 8.3–11.6)
Gender		
Male	6 (40%)	4 (26.7%)
Female	9 (60%)	11 (73.3%)
Handedness		
Right‐handed	10 (66.7%)	10 (66.7%)
Left‐handed	2 (13.3%)	1 (6.7%)
Ambidextrous	3 (20%)	4 (26.7%)
Full scale IQ	*M* = 110.3, SD = 6.7 (Range: 101.3–125.0)	*M* = 102.4, SD = 5.6 (Range: 92.5–115.0)

Children with DD were diagnosed using the Neuropsychological Test Battery for Number Processing and Calculation in Children—Revised (ZAREKI‐R) (von Aster et al. 2006). The diagnostic criteria were met if either the total score or the scores on three subtests were below the 10th percentile of the ZAREKI‐R. All participants were fluent Swiss German speakers and had normal or corrected‐toto‐normal vision. All participants agreed to participate, and written informed consent was obtained from all of their parents. The study was approved by the Ethics committee of Zurich, Switzerland, based on guidelines from the World Medical Association's Declaration of Helsinki (WMA 2013).

### 2.2 Behavioral Measures

#### 2.2.1 Numerical Abilities

Numerical abilities were assessed using the revised version of the Neuropsychological Test Battery for Number Processing and Calculation in Children (ZAREKI‐R) (von Aster et al. 2006). This test battery consists of 12 subtests assessing basic numerical skills and simple arithmetic (see Table S1 for detailed information about the subtests).

#### 2.2.2 Intelligence

Intelligence was estimated using the third edition of the Wechsler Intelligence Scale for Children (WISC) (Tewes et al. 1999) based on the subtests Similarities, Block Design, Vocabulary, and Picture Arrangement.

#### 2.2.3 Handedness

Handedness was determined by the Edinburgh Handedness Inventory (Oldfield 1971).

### 2.3 MRI

#### 2.3.1 fMRI Number Order and Number Identification Task

**FIGURE 1:**
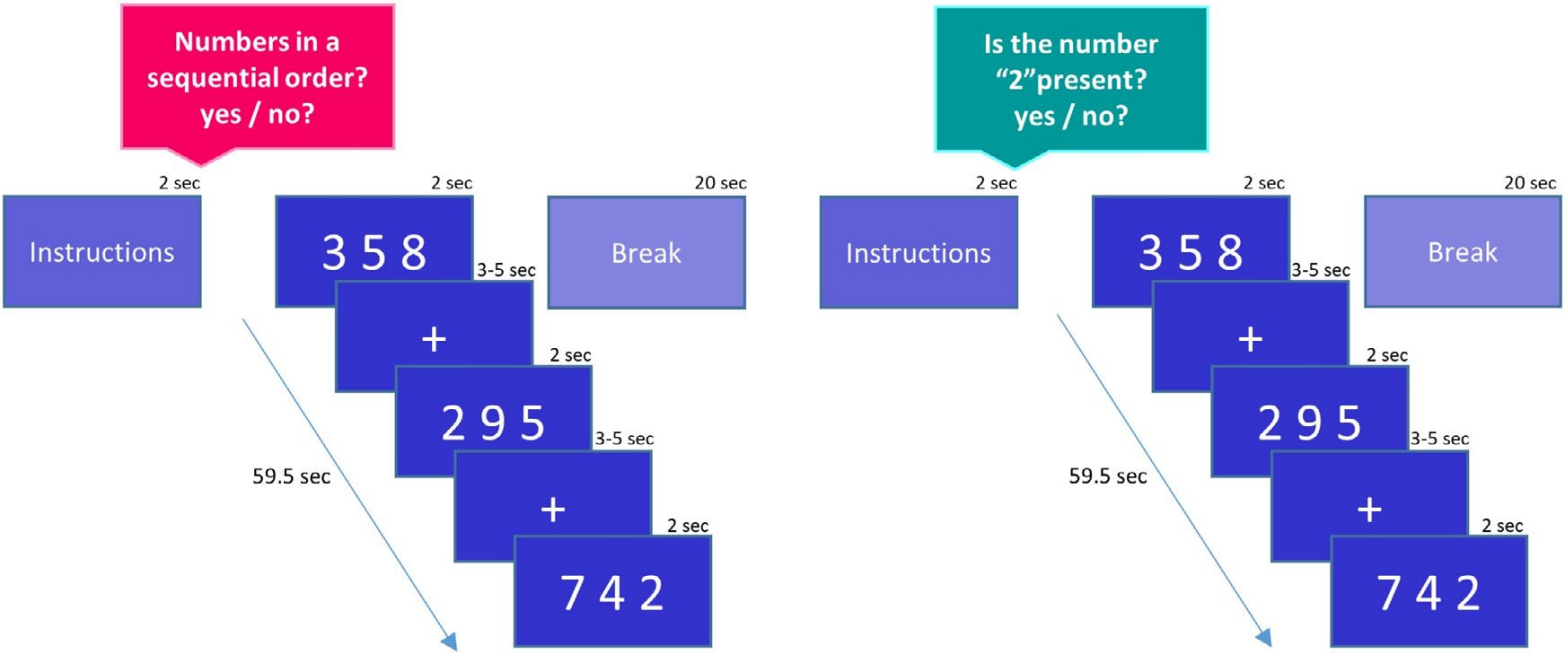
Schematic illustration of the fMRI task paradigm with totally eight blocks of number order and number identification alternatively presented. Each block contained 10 trials with a duration of 2 s, preceded by a short instruction (2 s) and followed by a break (20 s).

In the fMRI task, participants were presented with visual stimuli containing an array of three symbolic one‐digit numbers horizontally arranged using video goggles (VisuaStimDigital, Resonance Technology Inc., USA). The task contained two conditions: a number order and a number identification condition. In the number order condition, participants had to decide whether the three Arabic numbers appeared in a sequential manner (ascending or descending) by key press (yes or no). Using the same keys, in the number identification condition, participants had to decide whether the symbolic number “2” was present or not. The stimuli were presented in eight blocks of alternating condition; the interstimulus interval ranged from 3 to 5 s, and between the blocks was a resting period of 20 s. Before each block, the relevant task condition instruction appeared for 2 s. Each block contained 10 trials of the identical condition and had a total duration of 59.5 s, and the entire task lasted for 10.5 min; see Figure 1 for an overview of the paradigm.

All stimuli were built out of three Arabic digits between “2” and “9.” The number order condition contained in total 40 stimuli of number arrays, of which half (= correct) contained an array arranged in a sequential order (i.e., 10 stimuli in ascending order and 10 stimuli in reversed descending order) and in the other half of the stimuli, the numbers were mixed without the arrangement of a sequential order (= incorrect). The sequential order of the numbers in the ascending condition (e.g., 2‐5‐7) was reversed in the descending items (e.g., 7‐5‐2) and mixed for the non‐ordered stimuli (e.g., 5‐2‐7). The number identification condition contained in total 40 stimuli of number arrays, of which half of them contained the number “2” (= correct) and half of them did not (= incorrect). The entire paradigm was balanced for numerical distance [maximal(n)‐minimal(n): 5, 6, or 7] between the ascending and descending trials, as well as between the correct and incorrect trials. Furthermore, the majority of the sequentially ordered trials were unfamiliar sequences that are not part of the count list (e.g., 2‐3‐4) or a multiplication table (e.g., 2‐4‐6 or 3‐6‐9). There were only six trials which contained numbers of a multiplication table but of which one position was skipped (e.g., 2–4‐8 or 8–6‐2) and thus are assumed to be more familiar than the first category.

The task was programmed on E‐Prime (Version 2, Psychology Software Tolls Inc., USA) and participants' responses were recorded by an MRI‐compatible response box (Lumina Respond Pad, Cedrus Corporation, USA).

#### 2.3.2 Image Acquisition and Preprocessing

MRI data were acquired on a 3 T General Electric Signa Magnetic Resonance Imaging scanner (GE Medical Systems, USA) using an 8‐channel head coil. Whole brain functional images were acquired interleaved with a gradient echo EPI sequence [36 slices, slice thickness (ST) = 3.4 mm, no interslice skip, matrix size (MS) = 64 × 64, field of view (FOV) = 220 × 220 mm, in‐plane resolution = 3.4 × 3.4 mm, flip angle (FA) = 45°, echo time (TE) = 31 ms, repetition time (TR) = 2100 ms]. Structural images were collected using a T1‐weighted fast spoiled gradient echo sequence (3D FSPGR, number of slices = 172, slice thickness = 1 mm, no interslice skip, matrix size = 256 × 256, field of view = 240 × 240 mm, FA = 20°, TE = 3 ms, TR = 10 ms, TI = 500 ms).

Preprocessing of the images was conducted using Statistical Parametric Mapping (SPM12, Wellcome Trust Centre for NeuroImaging, UK, http://www.fil.ion.ucl.ac.uk/spm/) running on Matlab (Release 2017b, The MathWorks Inc., USA). Functional images were first slice‐time corrected and then realigned and unwarped. The resulting six motion parameters (translation in x, y, and z direction, rotation in pitch, roll, and yaw) were included later in the analysis to correct for motion. Next, the structural images were co‐registered to the mean‐functional images. Tissue probability maps matched for age and gender of our subjects sample were calculated using the CerebroMatic toolbox (Wilke et al. 2017). These tissue probability maps were then used to segment the co‐registered structural images and to estimate the normalization parameters, which were used to transform the functional images into the Montreal Neurological Institute (MNI) space. The normalized images were interpolated to a resolution of 2 mm^3^ and the functional images were smoothed with a Gaussian kernel of 6 mm FWHM (full width half maximum).

Finally, we calculated the displacement from scan to scan (STS) that reflects the movement between two consecutive volumes using the motion fingerprint toolbox (Wilke 2012). Applying a threshold as previously used (Binder et al. 2021) subjects with STS bigger than 0.5 mm in more than 20% of the volumes were excluded from statistical analyses. This resulted in the exclusion of two TD and three DD participants.

### 2.4 Data Analysis

#### 2.4.1 Behavioral Data

##### 2.4.1.1 Numerical Abilities

To compare the group in terms of percentile ranks achieved in the assessment of numerical abilities by means of the ZAREKI‐R, we conducted independent‐samples Mann–Whitney *U*‐tests. We compared the total ZAREKI‐R score to confirm our expectation that DD will demonstrate significantly lower percentile ranks. In a second step, we compared all the ZAREKI‐R subtests, where we Bonferroni adjusted the significance threshold to *p* = 0.0031 in order to correct for multiple comparisons. As outlined above, we expected that the DD group will perform significantly poorer compared to the TD group in arithmetic and symbolic and non‐symbolic number comparison (i.e., #4a addition; #4b subtraction; #4c multiplication; #8 oral number comparison; #9 non‐symbolic quantity estimation #12 symbolic number comparison, see Table S1). Due to the heterogenic nature of DD, we followed an exploratory approach for the remaining subtests.

##### 2.4.1.2 Behavioral Performance During fMRI Task

We conducted mixed design analyses of variance (ANOVA) to evaluate the effect of the within subjects factor “task condition” (number order, number identification) and the between‐subjects factor of “group” (DD, TD) once using the accuracy measure (percentage of correct answers out of total trials per condition) and once the reaction times (RTs) in seconds.

#### 2.4.2 MRI Data

##### 2.4.2.1 Statistical Analyses

At the single subject level, task effects were estimated using the general linear model (GLM) as implemented in SPM12. Time series of each subject were modeled with an event‐related design (i.e., modeling of all event onsets and durations) using a canonical hemodynamic response function (HRF). Both conditions (i.e., number order, number identification) were modeled as regressors of interest. Instruction trials, inter‐stimulus trials, and resting trials between the blocks entered the model as baseline. Time series were corrected for motion artifacts by entering the subject's six motion parameters as regressors of no interest. Finally, a high‐pass filter of 180 s was used to exclude slow signal drifts that occurred beyond this threshold.

In order to identify regions of interest that demonstrated an experimental effect across all participants, we conducted a second‐level one‐sample *t*‐test for the contrast images number order > number identification of all subjects from the first‐level analyses. This is because of our particular interest in including brain regions associated with number ordering. According to the guidelines (Zeidman, Jafarian, Corbin, et al. 2019), we applied a low threshold (i.e., *p* < 0.001 uncorrected) and discarded all regions below a cluster size of 10 voxels. Anatomical localization of the fMRI whole brain analyses results is reported in the MNI coordinate space; see Table S3.

##### 2.4.2.2 Dynamic Causal Modeling

Dynamic causal modeling (DCM) using SPM12 was applied to estimate the underlying neural activity of the fMRI data and the causal relation between brain regions (Friston et al. 2003; Zeidman, Jafarian, Corbin, et al. 2019; Zeidman, Jafarian, Seghier, et al. 2019).

###### 2.4.2.2.1 Region of Interest Time Series Extraction

The regions of interest (ROIs)—right anterior intraparietal sulcus (aIPS; 36, −46, 40 \[MNI]), right dorsal anterior insula (d‐aINS; 32, 16, 10), right pre‐supplementary motor area (preSMA; 6, 10, 50), right dorsal premotor cortex (dPMC; 26, 2, 54), right ventral premotor cortex (vPMC; 42, 0, 32), and vermis lobule VI (VER‐VI; 2, −70, −22)—were identified using a systematic two‐step approach that combined data‐driven analysis with theoretical considerations. We first identified task‐relevant regions based on the GLM contrast analysis (number order vs. identification) as mentioned above, then selected regions supported by spatial clustering and prior literature. The final set of ROIs reflected a trade‐off between theoretical relevance and data quality, aiming to retain the most informative combination of regions while maximizing participant inclusion—that is, ensuring all included participants showed activation in all selected ROIs. Nine participants had to be excluded because they did not show activation in all of the six ROIs (See Section S5 for detailed information).

**FIGURE 2:**
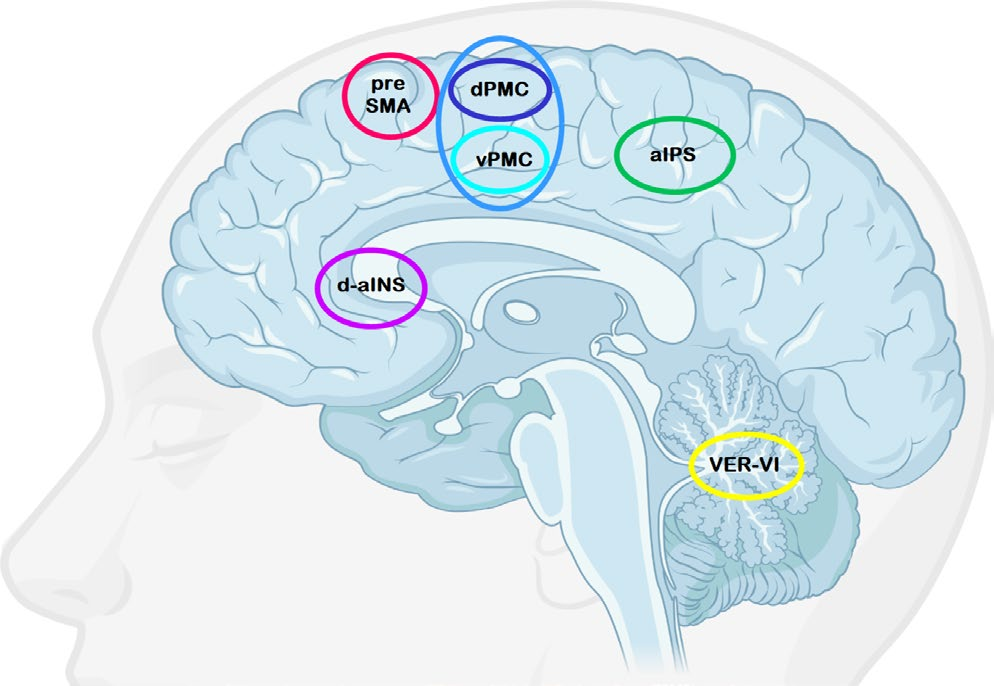
Regions of interests (ROIs) that defined the DCM model space. In total six ROIs in the right hemisphere were defined including anterior intraparietal sulcus (aIPS), dorsal and ventral premotor cortex (dPMC, vPMC), pre‐supplementary motor area (preSMA), dorsal portion of the anterior insula (d‐aINS) and vermis lobule VI (VER‐VI). The figure was created with BioRender.com.

For the purpose of extracting the time series, we created an effects of interest F‐contrast that included the two regressors number order and number identification as effects of interests and instruction, interstimulus and inter‐block break trials formed the baseline. We then used the SPM GUI “volume of interest” to extract the individual time series corrected for the motion parameters that corresponded to the principal eigenvariate with a center of 8 mm radius sphere within in a 8‐mm radius spherical search volume. The search volume was defined at *p* < 0.05 around the participants local maxima based on the coordinates of the activations detected in the number order > number identification contrast (Figure 2).

###### 2.4.2.2.2 DCM Model Specification

Our DCM model space was defined by a fully connected model which means that all possible bidirectional connections of extrinsic (i.e., between‐region) connectivity between the six ROIs were switched on. We implemented a one‐state, bilinear DCM with mean‐centered inputs (Friston et al. 2003), estimating the endogenous parameters (A‐parameters) that represent common effective connectivity shared across experimental conditions. Connectivity parameters between regions are expressed in Hz, with positive values indicating excitatory influence (i.e., increased activity in the source region increases activity in the target region) and negative values indicating inhibitory influence. Self‐connections represent a region's self‐inhibition, modeled as log‐scaling parameters around a default of −0.5 H. Positive self‐connection values indicate increased inhibition, and negative values indicate decreased inhibition. The task, comprised of all trials of the number order and number identification condition, was defined as the driving input for all the regions. Both conditions (i.e., number order, number identification) were defined as modulatory parameters (B‐parameter) on all the self‐connections and between connections, reflecting additive changes on the A‐matrix. This model was then specified and estimated for each participant. Following this procedure, one participant was excluded due to insufficient explained variance (4%).

###### 2.4.2.2.3 Parametric Empirical Bayes—Group Level Analyses

In order to estimate effective connectivity on a group level, we used the parametric empirical Bayes (PEB) framework (Friston et al. 2003; Zeidman, Jafarian, Seghier, et al. 2019). We specified a group covariate that was defined by coding the TD subjects with 0 and the DD subjects with 1. This means that the commonalities reflect the group average of the TD subjects, and the group covariate shows the changes by the DD group. Nuisance covariates (gender, age, handedness) were not included in the final model, as systematic model comparison revealed that the group‐only model provided the best fit (see Supporting Information).

We applied Bayesian model reduction (BMR) to identify the optimal model structure, as recommended for exploratory DCM analyses with large model spaces (six nodes). This procedure generates all possible parameter combinations with equal prior likelihood and iteratively prunes connections that do not contribute to model evidence, terminating when further parameter elimination decreases model evidence (Zeidman, Jafarian, Seghier, et al. 2019). This step was followed by a Bayesian model averaging (BMA) that averages the parameters (connection strength) from the best reduced models. This procedure we conducted separately for the A and B parameters.

For our interpretation of the outcome, we considered only the connectivity parameter with free energy that had a posterior probability greater than 99% based on the model evidence, which indicates a very strong evidence that this parameter is necessary to explain the data.

###### 2.4.2.2.4 Leave‐One‐Out Cross Validation With PEB

Finally, we tested whether the group effect size, which resulted from the previous analyses, was large enough for predictive validity (i.e., to predict a diagnosis of DD of a new subject) based on the modulation of effective connectivity by number order and number identification. For this purpose, we chose the connectivity parameters that had a threshold above 99% posterior probability (very strong evidence) to enter the leave‐one‐out validation analysis. In this procedure, a PEB model is fitted by leaving one subject out, and the estimations are used to predict the group allocation of the left‐out subject, which is repeated for every subject being left out. The predicted accuracy values are then correlated with the observed group allocation using point‐biserial correlation analysis to determine the accuracy of this prediction. The analyses were conducted based on the modulation by number order and number identification and separately for each of the modulation conditions (i.e., number order, number identification). Furthermore, we also tested whether each of the identified parameters of both modulating conditions would have predictive validity if considered individually.

## 3 Results

### 3.1 Demographic Characteristics

Participant demographic characteristics are presented in Table 1. Groups did not differ significantly in age (*t*(28) = 0.59, *p* = 0.558), gender distribution (*χ* ^2^(1) = 0.60, *p* = 0.439), or handedness distribution (Fisher's exact test, *p* = 0.357). Intelligence scores differed significantly between the groups (*t*(28) = −3.49, *p* = 0.002) with the TD group showing higher scores than the DD group. However, all participants demonstrated average or above‐average intelligence, with mean IQ scores above 85 in both groups.

### 3.2 Behavioral Results

#### 3.2.1 Numerical Abilities

We conducted independent‐samples Mann–Whitney *U*‐test to analyze group differences in the ZAREKI‐R total score and of each of the subtests. The groups significantly differed in the total ZAREKI‐R percentile rank, *U* = 212.0, *p* < 0.001 (TD mean rank 22.13, DD mean rank 8.87). Using the same statistical test, we further evaluated how groups differed in the ZAREKI‐R subtests, and these analyses showed significant differences in the subtests number writing, addition, subtraction, reading numbers, digit span backwards, oral number comparison, non‐symbolic quantity estimation, and story problems. After Bonferroni correction (*p* = 0.0031) only the subtests number writing, addition, subtraction, and non‐symbolic quantity estimation remained significant. No significant group differences were observed for the remaining ZAREKI‐R subtests. The statistical results of all ZAREKI‐R subtests are displayed in Table S2.

#### 3.2.2 Behavioral Performance on fMRI Number Order and Number Identification Task

**FIGURE 3:**
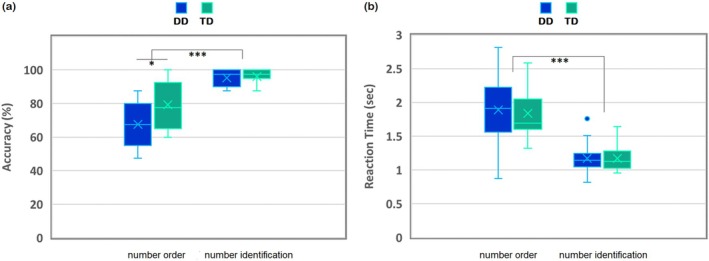
Accuracy in percentage (a) and reaction time (RT) in seconds (b) per condition (number order, number identification) and group (DD = children with dyscalculia, TD = typically developing children) during the fMRI task. Boxplots show the distribution of values across conditions and groups: The box represents the interquartile range (25th–75th percentile), the horizontal line indicates the median, the X marks the mean, and whiskers extend to the minimum and maximum values excluding outliers. Significant differences between group or conditions are illustrated with *p < 0.05 and ***p < 0.001.

Mixed design ANOVA was conducted using the measure accuracy in percentage to test the effects of within‐subjects factor condition (number order, number identification) and the between‐subjects factor group (DD, TD), see Figure 3a. The results showed that all three effects were significant: main effect of condition, *F*(1, 28) = 86.3, *p* < 0.001, *ηp* ^2^ = 0.48; main effect of group, *F*(1, 28) = 5.26, *p* = 0.029, *ηp* ^2^ = 0.16; and the interaction of group by condition, *F*(1, 28) = 5.33, *p* = 0.030, *ηp* ^2^ = 0.16. To understand the source of the significant interaction, we conducted independent *t*‐tests comparing the accuracy of each of the conditions between the groups, which revealed that groups significantly differed in the number order condition, *t*(28) = −2.39, *p* = 0.024 (DD: *M* = 67.5, SD = 13.8; TD: *M* = 79.3, SD = 13.4), but not the number identification condition, *t*(28) = −0.56, *p* = 0.578 (DD: *M* = 95.3, SD = 4.3; TD: *M* = 96.2, SD = 3.8). Paired sampled *t*‐test analyses revealed that accuracy in both conditions within the groups significantly differed: DD: *t*(14) = −8.03, *p* < 0.001; TD: *t*(14) = −5.05, *p* < 0.001. These results show that both groups performed better in the number identification compared to the number order condition. Furthermore, DD compared to TD performed significantly worse in the number order condition but did not differ significantly in the accuracy of the number identification condition.

The same analyses using reaction times (RTs) as measures (number control: DD: *M* = 1.9, SD = 0.48; TD: *M* = 1.8, SD = 0.38; number identification: DD: *M* = 1.2, SD = 0.23; TD: *M* = 1.2, SD = 0.19) revealed a significant main effect of condition, *F*(1, 28) = 140.80, *p* < 0.001, *ηp* ^2^ = 0.83 (see Figure 3b). RTs for the number order condition (*M* = 1.9, SE = 0.08) were significantly higher compared to the number identification condition (*M* = 1.2, SE = 0.04). The group main effect, *F*(1, 28) = 0.04, *p* = 0.838, *ηp* ^2^ = 0.002, and group by condition interactions, *F*(1, 28) = 0.20, *p* = 0.662, *ηp* ^2^ = 0.01, were not significant.

### 3.3 MRI Analyses

#### 3.3.1 Whole Brain fMRI Analyses

For the purpose of identifying brain regions showing an experimental effect in order to include them as ROIs in our DCM analyses, we conducted a one‐sample *t*‐test (thresholded at *p* < 0.001 uncorrected, *n* = 40) of all children with the number order > number identification contrast. As expected, this analysis identified a network of brain regions previously reported to be involved in numerical processing, including bilaterally the intraparietal sulcus, insula, pre‐supplementary motor area, middle cingulum cortex, dorsal premotor cortex, ventral premotor cortex, cerebellum (VI), right thalamus, and vermis. Although many regions were bilaterally activated, the network was dominated by right hemispheric activation. See Supplementary Table 3 for an overview of the activated brain regions and statistical results. Based on these activation results, we selected our regions of interest (see Section 2.4.2.2.1).

#### 3.3.2 DCM‐PEB Analyses

##### 3.3.2.1 Common Connectivity Across Conditions and Group Differences

**FIGURE 4:**
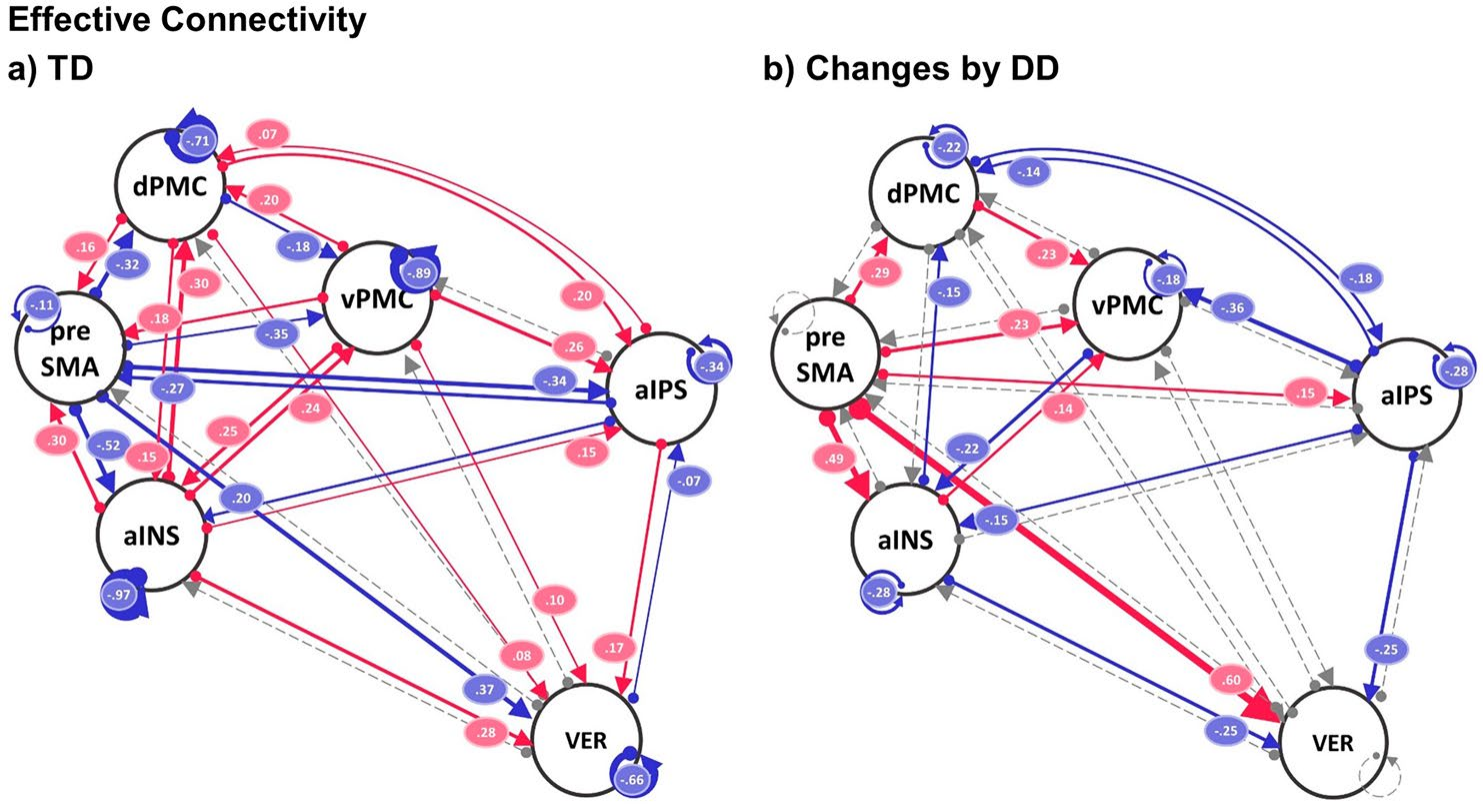
Dynamic causal modeling (DCM) results of common effective connectivity across task conditions (A‐matrix) for between‐connectivity and self‐inhibition of the ROIs for typically developing (TD) children and children with developmental dyscalculia (DD): (a) group mean of TD and (b) credible group mean differences by DD. Parameters with posterior probability different from zero and > 99% are color‐coded based on the direction of the effect (red = change toward positive direction, blue = change toward negative direction) and labeled with the corresponding parameter value of connectivity strength or log scales for self‐inhibitions. The thickness of the lines reflects the relative strength of connectivity. Non‐credible parameters and thus not necessary to explain the data are dashed‐lined and gray‐colored. aIPS, anterior intraparietal sulcus; dPMC, dorsal premotor cortex; vPMC, ventral premotor cortex; preSMA, pre‐supplementary motor area; d‐aINS = dorsal portion of the anterior insula; VER‐VI, vermis lobule VI.

Figure 4 shows the common effective connectivity across conditions for TD (left scheme) and the group effect by DD (right scheme) of the reduced model displaying only credible parameters with posterior probability > 0.99. During the model reduction, some parameters were removed since they were not necessary to explain the data. All parameter estimates and their associated posterior probabilities are displayed in Figure S2. Our main interest lies in the differences in the modulation of the common effective connectivity by the number order and number identification task conditions; therefore, we only describe briefly the outcome of the common connectivity across conditions. Overall, in TD, we can see that all outgoing connectivity of the preSMA was inhibitory, whereas all outgoing connectivity of the d‐aINS was excitatory. Most of the outgoing and incoming connectivity of the aIPS was excitatory. DD showed the strongest group effect in the connectivity from the preSMA to the d‐aINS and to the VER‐VI toward the positive direction. This resulted in a strongly decreased inhibitory connectivity from the preSMA to the d‐aINS and for the parameter preSMA to VER‐VI to a switch from inhibitory to excitatory connectivity. Moreover, DD compared to TD had decreased self‐inhibition for the aIPS, the dPMC, and vPMC, which shows that, in DD, these regions were more sensitive to the input from the network.

##### 3.3.2.2 Modulatory Effects on Common Connectivity and Group Differences

**FIGURE 5:**
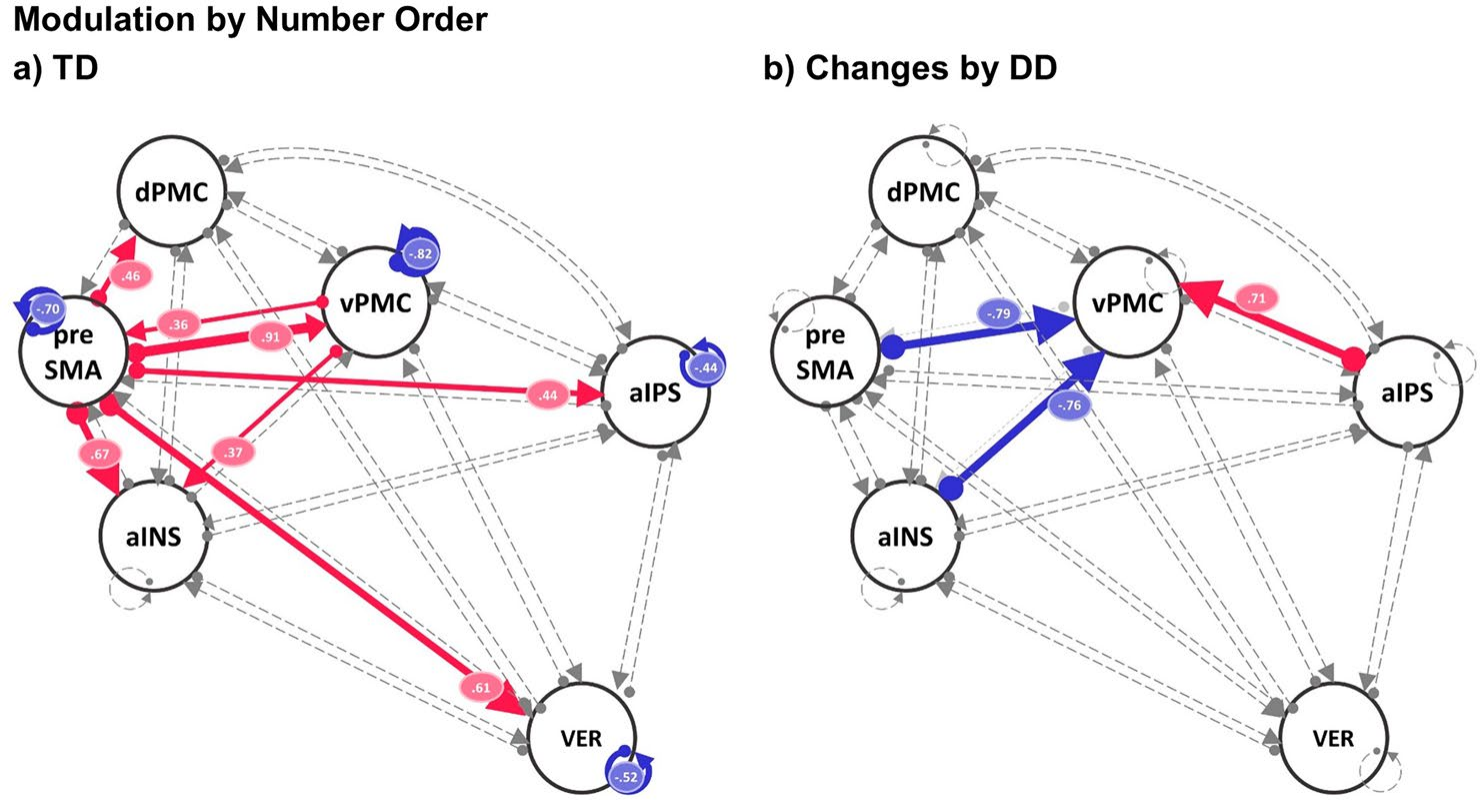
Dynamic causal modeling (DCM) results of the modulation (additive effect) by number order condition on common effective connectivity (B‐matrix) for between‐connectivity and self‐inhibition of the ROIs for (a) typically developing children (TD) and (b) changes by children with developmental dyscalculia (DD). Parameters with posterior probability of being different from zero of > 99% are color‐coded based on the direction of the effect (red = change toward positive direction, blue = change toward negative direction) and labeled with the corresponding parameter value of connectivity strength or log scales for the self‐inhibitions. The thickness of the lines reflects the relative strength of connectivity. Non‐credible parameters are dashed‐lined and gray‐colored. aIPS, anterior intraparietal sulcus; dPMC, dorsal premotor cortex; vPMC, ventral premotor cortex; preSMA, pre‐supplementary motor area; d‐aINS, dorsal portion of the anterior insula; VER‐VI, vermis lobule VI.

We found a strong modulatory effect of number order (see Figure 5) in TD on all outgoing connections of the preSMA. These effects were positive, which means that the relevant parameters switched from inhibitory to excitatory connectivity under the number order condition. In addition, outgoing excitatory connectivities from the vPMC to the d‐aINS and the preSMA were increased. Self‐inhibition of the aIPS, vPMC, preSMA, and the VER‐VI was strongly reduced and thus more sensitive for the input of the network. Taken together, during the number order condition, the network becomes in general more excitatory, leading to increased activity. We found strong group effects by DD toward a positive direction from the aIPS to the vPMC and toward a negative direction of the incoming connectivity to the vPMC from the d‐aINS and the preSMA. Thus, our results show that DD versus TD showed increased excitatory connectivity from the aIPS to the vPMC, strongly decreased excitatory connectivity from the preSMA to vPMC, and increased inhibitory connectivity (that had switched from excitatory to inhibitory) from the d‐aINS to the vPMC.

**FIGURE 6:**
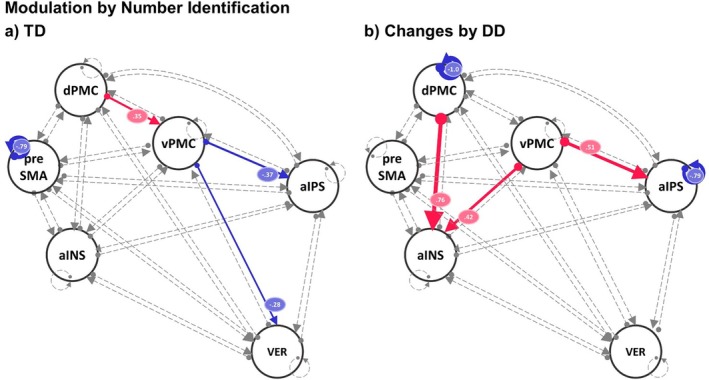
Dynamic causal modeling (DCM) results of the modulation (additive effect) by number identification condition on common effective connectivity (B‐matrix) for between‐connectivity and self‐inhibition of the ROIs for (a) typically developing children (TD) and (b) changes by children with developmental dyscalculia (DD). Parameters with posterior probability different from zero and > 99% are color‐coded based on the direction of the effect (red = change toward positive direction, blue = change toward negative direction) and labeled with corresponding parameter values of connectivity strength or log scales for self‐inhibitions. The thickness of the lines reflects the relative strength of connectivity. Non‐credible parameters are dashed‐lined and gray‐colored. aIPS, anterior intraparietal sulcus; dPMC, dorsal premotor cortex; vPMC, ventral premotor cortex; preSMA, pre‐supplementary motor area; d‐aINS, dorsal portion of the anterior insula; VER‐VI, vermis lobule VI.

For the condition number identification (Figure 6), we found TD modulatory effects toward the negative direction for the outgoing connectivity from the vPMC to the aIPS and to the VER‐VI (both switched from excitatory to inhibitory) and a positive modulatory effect for the connectivity from the dPMC to the vPMC (that switched from inhibitory to excitatory). Furthermore, the self‐inhibition of the preSMA was strongly reduced. The group effect of DD on the number identification modulation parameters went in a positive direction, which led to strongly increased excitatory connectivities from the dPMC to the d‐aINS, from the vPMC to the d‐aINS and from the aIPS to the vPMC. Moreover, in DD, self‐inhibition of the aIPS and dPMC was reduced and thus more sensitive to the input from the network for DD.

##### 3.3.2.3 Leave‐One‐Out Cross‐Validation

**FIGURE 7:**
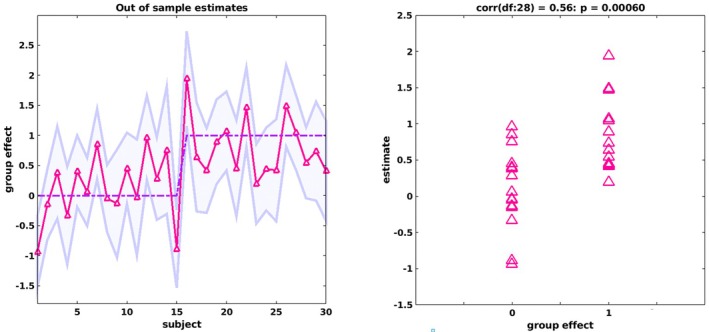
Leave‐one‐out cross‐validation predicting group effect (group allocation to DD or TD) of the modulation by both task conditions. Left: The out‐of‐sample estimates of group effect (red line) for each participant (i.e., participant 1–15 belong to TD, participant 16–30 to DD) with 90% confidence interval (shaded area) for each participant. The dashed red line represents the actual value of the group covariate Right: The correlation between actual group covariate value and the expected values for each participant.

**FIGURE 8:**
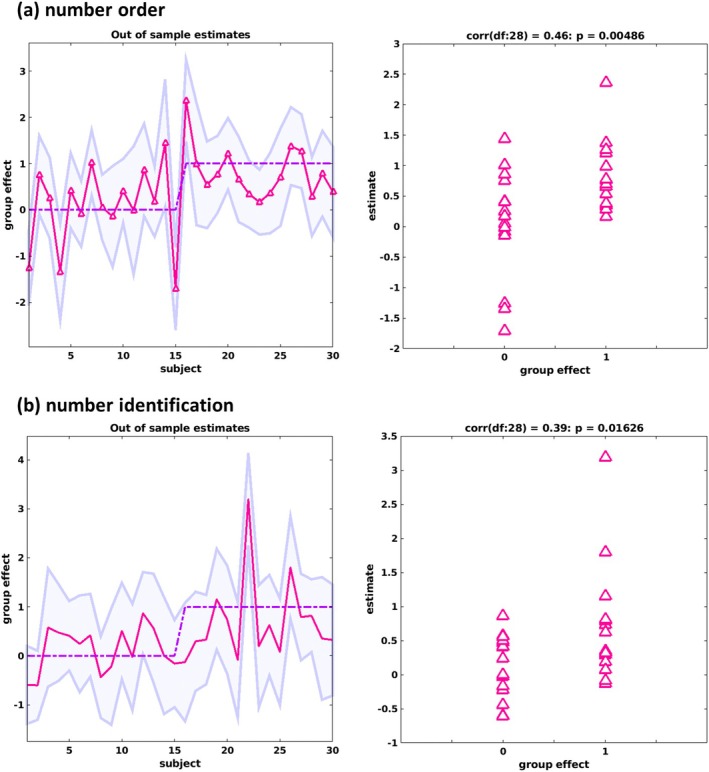
Leave‐one‐out cross‐validation predicting group effect (group allocation to DD or TD) of the modulation separately by number order (a) and number identification (b). Left: The out‐of‐sample estimates of group effect (red line) for each participant (i.e., participant 1–15 belong to TD, participant 16–30 to DD) with 90% confidence interval (shaded area) for each participant. The dashed red line represents the actual value of the group covariate Right: The correlation between actual group covariate value and the expected values for each participant.

In a leave‐one‐out cross‐validation (i.e., point‐biserial correlation analysis), the modulation of the effective connectivity by number order and number identification was found to significantly predict group allocation (*r*(28) = 0.56, *p* < 0.001) (Figure 7). In the DD group, 13 of 15 participants had their true group label within the predicted 90% confidence interval (mean CI width: 1.46, SD = 0.19) and 11 of 15 in the TD group (mean CI width: 1.41, SD = 0.32). When we analyzed the two modulators separately, it shows that the size of the group effect during number order modulation (*r*(28) = 0.46, *p* = 0.005) was larger (Figure 8a) compared to the modulation during number identification (Figure 8b) (*r*(28) = 0.39, *p* = 0.016) and that both were large enough to predict a subject's group allocation. For number order, the true label fell within the predicted CI for 13 participants with DD (Mean CI width: 1.78, SD = 0.30), and 10 TD participants (Mean CI width: 1.82, SD = 0.45). For number identification, the true label was within the predicted CI for 14 DD participants (Mean CI width: 2.10, SD = 0.29), and 14 TD participants (Mean CI width: 1.91, SD = 0.34). These results show that the highest predictive validity is obtained when parameters from both modulators are jointly considered, as indicated by the narrowest confidence interval, reflecting the most precise model estimation, and by the largest effect size, indicating the strongest group differentiation.

Finally, we tested which parameters with a credible group effect, when considered individually, predict group allocation with sufficient large effect sizes. Our analyses revealed that, during number order modulation, the between connectivity parameters from d‐aINS to vPMC, *r*(28) = 0.42, *p* = 0.011 (correct prediction: 12 DD; Mean CI width: 2.32, SD = 0.31, 10 TD; Mean CI width: 2.41, SD = 0.42), and from preSMA to vPMC, *r*(28) = 0.31, *p* = 0.048 (correct prediction: 15 DD; Mean CI width: 2.50, SD = 0.28, 13 TD; Mean CI width: 2.64, SD = 0.31) and self‐inhibition of the aIPS, *r*(28) = 0.36, *p* = 0.025 (correct prediction: 15 DD; Mean CI width: 3.23, SD = 0.13, 15 TD; Mean CI width: 3.21, SD = 0.20) during number identification, have large enough effect sizes to significantly predict group allocation. However, they did not survive Bonferroni correction of multiple comparison (*p* = 0.006). All remaining correlations were not significant.

## 4 Discussion

Number order has been shown to predict arithmetic skills (Lyons and Ansari 2015; Morsanyi et al. 2020; Sommerauer et al. 2020) and is together with arithmetic (Peters and De Smedt 2018) one of the abilities particularly impaired in children with developmental dyscalculia (DD) (Devlin et al. 2022; Morsanyi et al. 2018). However, little is known about the interplay of these regions during number processing, and no knowledge is available how these regions work together in children with DD. Therefore, we investigated in this study how brain regions associated with number order processing do interact functionally in children with DD and in typically developing children (TD) by applying effective connectivity analyses using dynamic causal modeling (DCM) (Friston et al. 2003). For this purpose, we analyzed fMRI data of children with and without DD who had performed on a symbolic number processing task with two conditions such as number order judgment and number identification. We evaluated both the modulatory effect of number order and number identification on the common effectivity across task conditions and the effect of group by DD.

On a behavioral level, we assessed a range of basic numerical abilities and simple arithmetic, which revealed that children with DD showed difficulties specifically in additions and subtractions as well as in non‐symbolic quantity estimation compared to TD children. These results confirm previous findings demonstrating that children with DD are in particular impaired in arithmetic (Peters and De Smedt 2018). Reports of impairment in non‐symbolic number processing in DD are inconsistent throughout literature, as pointed out by De Smedt et al. (2013) and usually deficits in symbolic number processing are more pronounced than in non‐symbolic number processing (Olsson et al. 2016). A likely explanation for this inconsistency is, as previously suggested by Rousselle and Noël (2007), and further elaborated by De Smedt et al. (2013), that deficits in non‐symbolic quantity processing become apparent only in later childhood (i.e., around 10 years of age) since they are preceded by symbolic number deficits which affect the refining of the approximate number system (ANS). Unexpectedly and against the current view in the literature, children with DD did not perform significantly worse in the symbolic number comparison task. This finding may be related to the special nature of the task used. Unlike other tasks used in the literature that usually are composed of one or two‐digit symbolic numbers (Olsson et al. 2016), the task in the current study was built from the comparison of multi‐digit symbolic number pairs with up to five digits. Therefore, this condition may have posed difficulties even in some of the TD children, especially the younger ones. This indeed was the case. When comparing the performance of TD children by dividing them into two groups—those who fully mastered the task versus those who did not—we found that the latter group was significantly younger than the former.

Our analyses of the behavioral data of the fMRI number order and number identification task revealed that performance on the number identification condition was similar for both groups in terms of accuracy and reaction time. The observation that, in the number identification condition, both groups reached ceiling effects and roughly used half of the time available indicates that the number identification task was easy for both groups to solve. Furthermore, in line with literature (Devlin et al. 2022; Morsanyi et al. 2018), children with DD performed significantly worse compared to TD children on the number order condition when analyzing accuracy. Although not directly assessed in this study, we presume that, for the number order task used in the current study, intact acuity of numerical representation is of particular relevance for successfully solving this task. This is because impaired acuity of numerical representation (i.e., impairment of the ANS and/or OTS) has been assumed to underlie the deficits shown during symbolic and non‐symbolic numerical processing (Olsson et al. 2016) and the acuity of the ANS has previously been related to number order ability (Lyons and Beilock 2011). Moreover, the speed component of the used task may have amplified the significance of the acuity of the ANS for performance on the number order condition. This suggests that ANS impairments may exert a greater influence on number order ability under time‐limited conditions. Finally, the finding of the link that ANS acuity is related to number order ability and through that related to arithmetic (Lyons and Beilock 2011) might be reflected by our behavioral results, given that children with DD in particular were impaired in number order ability and arithmetic.

On the brain level, our analyses aiming to identify activations common across all children in the number order versus number identification contrast revealed a right hemisphere dominant brain network of right and left hemispheric regions in the anterior inferior and posterior parietal cortex, anterior insular cortex, lateral and medial frontal cortex as well as in the cerebellum. This brain network includes mainly brain regions previously identified in the context of number processing (Arsalidou et al. 2018). Out of this network, we defined six regions of interest that were most consistently activated in the majority of our children, which were all located in the right hemisphere, including the pre‐supplementary motor area (preSMA), the dorsal (dPMC) and ventral premotor cortex (vPMC), the anterior intraparietal sulcus (aIPS), the dorsal anterior insula (d‐aINS), and the cerebellar vermis lobule VI (VER‐VI). In terms of effective connectivity, we identified a range of parameters that distinguished DD from TD regarding their common effective connectivity across task conditions, which clearly indicates that DD differ from TD in the way their brain interacts within this network. The two parameters that were most affected by DD were the inhibitory between connectivity from the preSMA to the d‐aINS and to the VER‐VI, which was strongly decreased for the former and the latter switched from an inhibitory to an excitatory connectivity. Both the d‐aINS and the VER‐VI are strongly involved in salience detection (Molnar‐Szakacs and Uddin 2022; Habas 2022). A possible interpretation of this finding is, by taking into account the significant role of the preSMA in cognitive control (Obeso et al. 2013), that salience processing was increased in DD via these connectivities because of a poorer and less automatized numerical representation, which is common in children with DD (Olsson et al. 2016). In other words, it is likely that if numbers are less precisely represented, symbolic number stimuli do per se appear more salient. Alternatively, this group effect may also be related to a higher demand on affective regulation as the used task may pose a higher emotional stress to DD compared to TD. In TD, self‐inhibition of the preSMA and the aIPS was below the default value, which indicates that these regions were specifically sensitive for the input from the network. In DD, self‐inhibition was decreased in both premotor regions, the d‐aINS and the aIPS, which shows that, in DD, these regions were more sensitive to inputs from the network compared to TD. The reduction of self‐inhibition has been described in other disorders as a pathological feature of the imbalance between excitation and inhibition (Snyder et al. 2021).

The modulation of the common effective connectivity by number order and number identification is of our main interest and the related findings we discuss in detail in the following sections.

### 4.1 Modulation of Effective Connectivity by Number Order

In TD, number order modulated the common effective connectivity most notably by all outgoing connections from the preSMA toward a positive direction, resulting in a change from inhibitory to excitatory connectivity for all of these parameters. This indicates that the preSMA plays an important role in number order performance. The current finding aligns with previous research highlighting the preSMA's critical involvement in cognitive control and sequential processing (Cona and Semenza 2017; Nachev et al. 2008; Nakajima et al. 2022; Obeso et al. 2013, 2017; Roberts and Husain 2015; Tabu et al. 2011; Wolpe et al. 2022) and it further advances our understanding by demonstrating that the preSMA may dynamically coordinate task‐relevant neural interactions during numerical sequence processing. Moreover, given that the sequentially ordered number trials used in the current study contained unfamiliar sequences, it is unlikely that participants relied on memory retrieval. Instead, performance likely involved sequence learning processes. The preSMA has indeed been associated with sequence learning rather than sequence recall. This has been shown by a TMS study (Shimizu et al. 2020) showing that virtual lesion over preSMA led to a higher error rate during visuomotor sequence learning but not during sequence recall. Furthermore, in the context of motor sequence learning, Ohbayashi (2021) concluded in his review that the preSMA is primarily involved in the cognitive aspect of learning a new sequence. Processing of sequential relations between numbers appears as a core ability central to the solving of number order tasks. Comparing the magnitude of numbers is one type of strategy that has been suggested (Devlin et al. 2022) for unfamiliar sequences (e.g., 5‐7‐9) that are not part of the counting list (e.g., 1‐2‐3) or of multiplication tables (e.g., 2‐4‐6, or 3‐6‐9), as it was the case for the task used in the current study. To do so, executive functions are needed to switch between the numbers and to update the relevant information, which appears to be operated by the preSMA in concert with other regions involved. Hence, our results demonstrate that the preSMA has an orchestrating role directing the activation of all other regions involved in number ordering and therefore supports previous reports associating the preSMA with sequence learning and cognitive control (Cona and Semenza 2017; Nakajima et al. 2022).

The preSMA's role in sequence processing is further supported by findings from musicians, who show greater cortical thickness in the right preSMA compared to non‐musicians (Shenker et al. 2022). That study also reported increased thickness in the right dPMC and vPMC. As sequence processing is central to music production (Brown and Palmer 2012), pianists exhibit enhanced explicit and implicit sequence learning that cannot be explained by superior motor coordination alone (Schwizer Ashkenazi et al. 2022). These results highlight the critical role of the preSMA, dPMC, and vPMC in sequential processing—consistent with our finding of strongest modulation in the preSMA–vPMC connection. The specific contributions of these regions remain to be clarified, though a general distinction has been proposed: lateral premotor areas (vPMC, dPMC) respond more to external cues (e.g., visually presented numbers), while medial regions (preSMA, SMA proper) are more engaged by internally guided sequences (e.g., mentally represented number sequential relations) (Crosson et al. 2001; Schönberger et al. 2013).

The connectivities from the preSMA to d‐aINS and to VER‐VI were the second most strongly affected by number ordering. Both the d‐aINS and VER‐VI have been reported to be involved in salience detection (Habas 2022; Habas et al. 2009; Harsay et al. 2012; Taylor et al. 2012; Touroutoglou et al. 2012) and in cognitive flexibility (Berger et al. 2005; Menon and Uddin 2010; Ravizza and Carter 2008; Woolley et al. 2008). Thus, our finding may imply that the discrimination of whether the number arrays are arranged in a sequential order or not is likely supported by salience detection. Furthermore, cognitive flexibility is highly relevant in this task, since it demands fast switching between the numbers (i.e., max. 2 s available), which may explain the excitation of the d‐aINS and VER‐VI by the orchestrating region preSMA.

In addition, self‐inhibition was decreased in the aIPS, preSMA, vPMC, and VER‐VI, and these regions were, therefore, more sensitive during the number order condition to the input from the network, which shows the importance of these regions for the number order network.

Taken together, within the confines of our selected DCM network, our results indicate that successful performance on the number order task—particularly when involving unfamiliar sequences and time constraints—may rely in particular on the involvement of the right aIPS, preSMA, vPMC, and VER‐VI. In this network, the right preSMA appears to play a central role in modulating the involvement of other regions. Its strong connectivity with the right vPMC could reflect the recruitment of visuo‐spatial resources (Hoshi and Tanji 2007; Schubotz and von Cramon 2002) relevant for number representation. Additionally, modulations from the preSMA to the d‐aINS and VER‐VI may point to the engagement of salience processing and cognitive flexibility during task performance.

#### 4.1.1 Group Effect by DD on Modulation by Number Order

The group effect by DD on the modulation by number order revealed that connectivity from the aIPS to the vPMC was increased toward a positive direction and therefore excitatory. In contrast, connectivity from the d‐aINS to the vPMC was strongly modulated toward a negative direction (strongly inhibited) and the excitatory connectivity from preSMA to the vPMC was strongly decreased.

In the context of sensorimotor integration, the right vPMC has been proposed as a finger counting region, where canonical handshapes for numbers may become abstract representations (Krinzinger et al. 2011). Alternatively, the activation may reflect visuo‐spatial, visuo‐motor, and sequential processing, all associated with the right vPMC (Hoshi and Tanji 2007; Schubotz and von Cramon 2002). In Krinzinger et al.'s visually guided finger task, children pressed a finger in response to a red‐lit circle in one of four positions, requiring stimulus–response mapping, sequence learning, and visuomotor coordination (Vakil et al. 2017). Similarly, their non‐symbolic number tasks involved spatially distributed dot sets, likely engaging visuospatial and sequential processes.

Thus, activation in the vPMC may reflect both numerical representation via finger patterns and general sequence‐related visuomotor processes. Supporting this, premotor activation has been linked to handedness in finger counting (Tschentscher et al. 2012), and children with DD have been shown to persist in finger counting strategies beyond typical developmental stages (Price and Ansari 2013). Additional bilateral vPMC activation during sequential counting of visual and auditory stimuli has also been observed in a right‐lateralized frontoparietal network (Piazza et al. 2006).

Accordingly, the excitatory aIPS–vPMC connectivity may reflect stronger engagement of a finger counting and sequence‐processing network. In contrast, the d‐aINS–vPMC connectivity shifting from excitatory to inhibitory might indicate regulatory modulation in response to this recruitment. Notably, while preSMA–vPMC connectivity was strongly excitatory in TD children, it became negligible in DD during number order processing.

Overall, the modulation pattern in DD during number order processing points to an aberrant effective connectivity, which may be a reflection of their behavioral deficits in this task. However, considering the finding of Park et al. (2014) showing that the strength of effective connectivity from the right superior parietal cortex to the right PMC was related to math performance in very young children (i.e., 4 to 6 years old), we cannot exclude the possibility that this pattern is at least partly related to an immature network. In other words, the question of whether this pattern is primarily due to aberrant brain connectivity or a delay in the maturation of the number processing network cannot be answered in the current study and should be explored in future research.

### 4.2 Modulation by Number Identification

In TD, outgoing connectivity from the dPMC to vPMC had an effect toward a positive direction which resulted in a switch from an inhibitory common connectivity to excitatory connectivity during number identification, whereas the outgoing connectivities from the vPMC to the aIPS and VER‐VI switched from excitatory to inhibitory connectivity. This pattern indicates that the excitatory effect of the dPMC on the vPMC led to the inhibitory influences on the aIPS (associated in particular with numerical representation), and inhibition of the VER‐VI (associated with salience detection and cognitive flexibility).

The number identification condition required participants to focus solely on a specific number (i.e., no. “2”) as the target and ideally to suppress all the other numbers (distractors). Thus, our finding may reflect the implementation of such strategies. This is suggested, first, by the observed inhibition of regions such as the aIPS, typically active during number processing. In fact, number processing is not required to successfully solve this task and may only lead to noise. Second, the inhibitory influences on the VER‐VI may point to a downregulation of salience detection or stimulus switching processes (i.e., cognitive flexibility) which could otherwise interfere with the rapid and efficient solving of this task. Rather the opposite could potentially play a more relevant role for this task, namely a rigid focus on the given target number. Rigid cognitive behavior has indeed been observed in children with cognitive cerebellar affective syndrome who demonstrate damage in the vermis (Starowicz‐Filip et al. 2022). Furthermore, the finding that this modulatory connectivity is directed by the dPMC via the vPMC may go along with the established finding that the premotor cortex is in particular involved in the suppressing of incorrect response prior to a reaction (i.e., NoGo stimuli) (Falkenstein et al. 1999; Xia et al. 2020). The reduced self‐inhibition in the preSMA further underscores the importance of cognitive control for the solving of this task.

#### 4.2.1 Group Effect by DD on Modulation by Number Identification

The effect of DD on the modulation by number identification showed interesting and relevant findings. Contrary to TD, that presumably showed inhibition of numerical processing, as indicated by a switch from an excitatory to inhibitory connectivity from the vPMC to the aIPS, in DD, it appeared to be the opposite; DD showed increased connectivity (excitatory) likely implicating increased rather than suppressed numerical processing. At the same time, both premotor regions (dPMC, vPMC) had an excitatory group effect on the d‐aINS. The picture emerging is that although children with DD did not express behavioral impairment, on the neuronal level, they may be impaired in inhibiting numerical processing for a task containing numerical material without the need of numerical processing. This could possibly contribute to the observed increase in excitatory connectivity from both premotor regions to the d‐aINS, potentially reflecting greater salience processing and/or filtering (Molnar‐Szakacs and Uddin 2022). This might be associated with reduced precision in neuronal number representation, leading to neuronal noise that necessitates enhanced filtering and/or salience detection to compensate. A recent study by Barretto‐García et al. (2023) provides evidence for this assumption by showing that sharper neuronal numerosity representations, indicated by narrower BOLD signal variability in parietal numerosity‐tuned regions (Harvey et al. 2013), are associated with better performance on a non‐symbolic magnitude comparison task. In other words, the more precise the neuronal representation of numerosity tuned neuronal populations in the parietal cortex, the higher were the scores in the magnitude task. Thus, it is possible that on the neuronal level, children with DD may have a more diffuse representation of numerosity and therefore decreased ability to discriminate between numbers, which in turn might hamper the adequate suppression of distractors. Consequently, it is likely that even if behavioral task performance is not impaired, on the neuronal level, it appears more costly for DD, and eventually behavioral deficits may become apparent in the performance on higher demanding versions of such type of tasks.

Furthermore, the observed reduction in self‐inhibition in the aIPS and dPMC in DD, which implies increased sensitivity to network input, may lend further support to our assumptions. In particular, the decreased self‐inhibition of the aIPS indicates the involvement of numerical processing. This may suggest that the previously hypothesized increased processing of numbers in DD versus TD during number identification may also be related to an impaired neuronal balance for adequate filtering of numerical input, in particular for task‐irrelevant stimuli, as it has been found in attention deficit (hyperactivity) disorders (ADHD). For example, an fMRI study (Tegelbeckers et al. 2015) found that children and adolescents with ADHD failed to neuronally suppress the processing of novel but task‐irrelevant stimuli, as they were observed to allocate significantly more neural resources compared to TD children and adolescents. Moreover, the ADHD group failed to express neuronal habituation to familiar stimuli. Similarly, in an electroencephalography (EEG) study, Godefroid and Wiersema (2017) found increased sustained processing of novel but task‐irrelevant stimuli in adults with ADHD. In children with DD, these impairments may be related only to numerical stimuli since attention deficit disorders were part of the exclusion criteria for this study.

Moreover, considering that the right dPMC has been associated with rhythmic processing, explicit sequence learning, and encoding of difficulty (Giovannelli et al. 2014; Kantak et al. 2012; Mione et al. 2020), the decreased self‐inhibition of the dPMC may also indicate that, on a neuronal level, children with DD processed sequential relations between numbers. Given that this task condition does not rely on processing sequential number relations, in contrast to the number order conditions, the finding might point to a reduced neuronal cognitive flexibility, possibly leading to interference or noise between task conditions.

### 4.3 Integrating Effective Connectivity Findings With Functional and Structural Connectivity Literature in DD

Previous studies using functional connectivity analyses (e.g., Rosenberg‐Lee et al. 2015; Michels et al. 2018) have reported hyperconnectivity in children with DD, particularly between the IPS and frontal regions during numerical tasks. These findings suggest that difficulties in number processing in DD may stem more from inappropriate task modulation and hyperconnectivity than from under‐engagement or insufficient connectivity. Our effective connectivity results extend this literature by showing that such alterations are not only present at the level of undirected coactivation but also manifest as task‐dependent, directional changes in information flow. While functional hyperconnectivity reflects statistical synchrony, our findings suggest that the underlying mechanism may involve disrupted top‐down modulation (i.e., as likely indicated by the findings related to the number order modulation) or impaired filtering of numerical information, even in tasks where behavior is preserved. This highlights that hyperconnectivity in DD may not merely be a compensatory pattern but could reflect more fundamental alterations in the dynamics of cognitive control and numerical processing.

Moreover, our results converge with (micro‐)structural imaging findings, particularly diffusion tensor imaging (DTI) studies that have reported reduced white matter integrity (i.e., lower fractional anisotropy) in the superior longitudinal fasciculus (SLF) in individuals with DD (Rykhlevskaia et al. 2009; Kucian et al. 2014). Given the SLF's critical role in connecting parietal and frontal regions—including IPS, premotor cortex, and preSMA—the microstructural disruptions observed in DTI studies may underlie or contribute to the aberrant effective connectivity patterns we identified. In this context, our findings provide a possible mechanistic link between white matter alterations and altered network dynamics in DD.

Our findings indicate that number processing difficulties in DD are linked not only to functional hyperconnectivity and structural white matter abnormalities but also to disrupted task‐specific modulation of information flow. This may point to dysfunctional network dynamics as a potential mechanism contributing to the deficits observed in DD.

## 5 Limitations and Future

This study lacks the assessment of strategy use, in particular for the number order task condition. It is possible that DD and TD may not have used the same strategies, and a better understanding of behavioral mechanisms may have further complemented our findings. Generally, the literature lacks insights into strategies for solving non‐familiar number sequence order tasks (Devlin et al. 2022) and future studies should include the assessment of strategy use. In reference to the number identification task condition, we do not believe that there was much room for the use of different strategies on the behavioral level, based on the observation that both groups reached ceiling effects in accuracy and similar mean and standard deviations of reaction time. Nevertheless, we cannot exclude the possibility that the groups differed in their strategy use during the number identification condition, which may have influenced the outcome. Moreover, the number of participants in this study was at the lower limit of adequacy. Especially in light of the potential impact of individual strategy use, future studies should aim for a larger sample that allows for subgrouping based on strategy or other relevant behavioral variables. That said, our dynamic causal modeling (DCM) approach is less reliant on large sample sizes, as it estimates parameters within a Bayesian framework. Conservative priors and stringent thresholds were applied, and model robustness was further supported by leave‐one‐out cross‐validation at the group level. These measures support the credibility of the findings despite the modest sample size. Finally, gender was not balanced between groups, reflecting the higher prevalence of DD in females compared to males (Schulz et al. 2018). As a result, we were not able to reliably assess potential gender effects on effective brain connectivity. Additionally, we compared models with and without nuisance covariates (age, gender, and handedness) and found that the model with the highest free energy—indicating the best trade‐off between model fit and complexity—was the one without any covariates. Future studies with larger and more gender‐balanced samples are needed to clarify the potential influence of gender and other demographic variables on effective connectivity patterns in numerical processing in DD. Our work aimed to investigate, in an exploratory approach, global differences in causal connectivity in TD and DD related to the processing of number order and number identification in a right hemispheric brain network including parietal, frontal, insular, and cerebellar regions by following a full DCM model approach. Future studies may be encouraged to test specific hypotheses, such as the ones generated in our study. Moreover, it may be of great interest to examine effective connectivity of neural activity underlying other types of numerical tasks, including magnitude comparison or arithmetic, as well as contrasting number order tasks with non‐numerical order tasks, for example, using letters or pictures illustrating annual events (Morsanyi et al. 2018). Traditional fMRI analyses have revealed a large body of knowledge about which brain regions are involved in numerical processing. However, it may be of great importance to better understand the interaction between these regions in order to be able to address specifically the relevant impaired subfunctions during training sessions. Effective connectivity analyses using DCM may have the potential to contribute to critical insights necessary for the development of such targeted interventions.

## 6 Conclusion

An interesting finding of our study is that in the number identification task condition, which is considered behaviorally easy to solve and did not demonstrate any behavioral group differences, several group differences in effective connectivity were observed. Modulation of number identification in DD led to strong increased (excitatory) connectivity in several parameters (dPMC to d‐aINS, vPMC to d‐aINS, vPMC to aIPS), and moreover, self‐inhibition was reduced in the dPMC and the aIPS, meaning that these regions were more sensitive to the input of the network. This pattern may indicate additional recruitment of salience detection and numerical processing mechanisms that are not necessary for solving this type of task and could, in more demanding contexts, introduce neuronal noise that interferes with task performance. This may reflect reduced precision of neuronal number representation, which is compensated for by increased engagement of salience detection, integrative processing, and cognitive control mechanisms. Hence, these results may be taken as an indication of the combination of both domain‐specific and domain‐general processes underlying deficits of DD. Furthermore, this study demonstrates the expected finding that children with DD are impaired in the performance of a non‐familiar number order task. In addition, effective connectivity analyses showed an orchestrating role of the preSMA, probably linked to cognitive control processes involved in learning sequences and combining numerical information. DD showed strong differences compared to TD in incoming connectivity to the vPMC from the aIPS, the preSMA, and the d‐aINS between TD and DD. This pattern could be a sign of atypical network functioning in DD when processing number order. However, as mentioned above, we cannot exclude that this pattern is also related to a possible immaturity of the network (Park et al. 2014). By employing effective connectivity analyses using the DCM‐PEB framework, our study advances previous findings of aberrant functional and structural connectivity in DD, revealing for the first time the task‐dependent dysfunctional network dynamics that may underlie number processing deficits in DD.

## Author Contributions

**Simone Schwizer Ashkenazi:** conceptualization, methodology, data curation, formal analysis, visualization, writing – original draft, writing – review and editing. **Ursina McCaskey:** investigation, writing – review and editing. **Ruth O'Gorman Tuura:** writing – review and editing. **Karin Kucian:** supervision, writing – review and editing.

## Conflicts of Interest

The authors declare no conflicts of interest.

## Supporting information


Data S1.

## Data Availability

Data not available due to ethical restrictions.
