# Supplementary material for: Altered Effective Connectivity of the Numerical Brain in Children With Developmental Dyscalculia
Source: J Neurosci Res. 2025 Jul 21;103(7):e70066. doi: 10.1002/jnr.70066 (PMC12278192; doi:10.1002/jnr.70066)
Supplement: Supplementary file 1 — Data S1. [file JNR-103-e70066-s001.docx]

**Supplementary Material**

**Altered Effective Connectivity of the Numerical Brain in Children with Developmental Dyscalculia**

Simone Schwizer Ashkenazi^12^, Ursina McCaskey^13^, Ruth O’Gorman Tuura^134^, Karin Kucian^135^

^1^ Center for MR-Research, University Children’s Hospital Zurich, Zurich, Switzerland

^2^ Neuropsychology, Dept. of Psychology, University of Zurich, Zurich, Switzerland

^3^ Children’s Research Center, University Children’s Hospital Zurich, Zurich, Switzerland

^4^ Zurich Center for Integrative Human Physiology, University of Zurich, Zurich, Switzerland

^5^ Neuroscience Center Zurich, University of Zurich and Swiss Federal Institute of Technology Zurich, Zurich, Switzerland

Corresponding author:

Simone Schwizer Ashkenazi

Center for MR Research, University Children's Hospital of Zurich, Zurich, Switzerland

E–mail: Simone.Schwizer@gmail.com.

1. **Neuropsychological Test Battery for Number Processing and Calculation in Children – Revised (ZAREKI-R)**

The ZAREKI-R test battery is a norm-based test conceptualized to identify children with deficits in number abilities from grades 1 to 4. The ZAREKI-R consists of 12 subtests (see Supplementary Table 1) assessing basic number skills as well as mental calculation (arithmetic). Each correct answer is scored with 1 to 2 points resulting in a total raw test score of maximum 122 points. The test has no time limit. Criteria for DD are met if the scores in three subtests of the same factor or the total test score is below the 10th percentile rank.

**Supplementary Table 1**: Description of the 12 subtests of the Neuropsychological Test Battery for Number Processing and Calculation in Children – Revised (ZAREKI-R)

| No. | Subtest | Description | Scoring | Max. Points |
| --- | --- | --- | --- | --- |
| 1 | Enumeration | Enumeration of different sets of dots (e.g. 13 dots arranged in a line, 18 dots randomly arranged) | 1 point for each correct answer | 5 |
| 2 | Counting backwards | Counting backwards starting from a given number (e.g. 22-1, 67-54) | 2 points = no mistakes, 1 point = one mistake, no points < one mistake | 4 |
| 3 | Writing numbers | Writing Arabic numbers from dictation (e.g. 14, 4658) | 1 point for each correct answer | 8 |
| 4a | Arithmetic Addition | Solving of mental calculations: orally presented addition problems | 1 point for each correct answer | 8 |
| 4b | Arithmetic Subtraction | Solving of mental calculations: orally presented subtraction problems | 1 point for each correct answer | 8 |
| 4c | Arithmetic Multiplication | Solving of mental calculations: orally presented multiplication problems | 1 point for each correct answer | 6 |
| 5 | Reading numbers | Reading aloud of written Arabic numbers (e.g. 305, 969) | 1 point for each correct answer | 8 |
| 6a | Number line with guidelines | Choosing correct position of visually or orally presented number out of four possible positions on a line from 1 to 100. | 1 point for each correct answer | 6 |
| 6b | Number line without guidelines | Positioning of visually or orally presented numbers by marking the position on a line from 1 to 100 | depending on distance to correct position the answer 1 or 2 points | 12 |
| 7a | Digit span forwards | Repeating of orally presented number sequence in the same direction | 1 point for each correct answer | 12 |
| 7b | Digit span backwards | Repeating of orally presented number sequence in the reverse direction | 1 point for each correct answer | 12 |
| 8 | Oral number comparison | Identifying the higher number out of orally presented number pairs (e.g. eight hundred – hundred and eight) | 1 point for each correct answer | 8 |
| 9 | Non-symbolic quantity estimation | Quantity estimation of visually presented sets of objects (e.g. 57 balls, 89 cups) | 1 point for each correct answer | 5 |
| 10 | Contextual quantity estimation | Quantity estimation in relation to the context if a quantity represents low, average or high quantity (e.g. fifteen words in a reading book, four fridges in a kitchen) | 1 point for each correct answer | 6 |
| 11 | Story problems | Solving of different story problems (e.g. Peter has 16 marbles. He has 4 marbles more than Anne. How many marbles does Anne have?) | 1 point for each correct answer | 6 |
| 12 | Symbolic number comparison | Identifying the larger number of visually presented multi digit Arabic number pairs (e.g. 654 or 546) that contained between two to five digits | 1 point for each correct answer | 8 |

1. **Basic numerical abilities and simple arithmetic in TD children and children with DD**

**Supplementary Table 2**. Mann-Whitney U Test statistics of ZAREKI-R subtests' accuracy percentile ranks

| No. | ZAREKI-R Subtests | Mean rank TD | Mean rank DD | *U* | *z* | *p* |
| --- | --- | --- | --- | --- | --- | --- |
| 1 | Enumeration | 16.5 | 14.5 | 127.5 | 0.89 | .373 |
| 2 | Counting backward | 17.2 | 13.8 | 138.0 | 1.43 | .154 |
| 3 | Writing numbers | 19.6 | 11.4 | 174.0 | 2.95 | **.003** |
| 4a | Arithmetic Addition | 21.1 | 9.9 | 196.0 | 3.61 | **<.001** |
| 4b | Arithmetic Subtraction | 21.9 | 9.1 | 208.0 | 4.02 | **<.001** |
| 4c | Arithmetic Multiplication | 15.3 | 12.4 | 109.5 | 1.31 | .191 |
| 5 | Reading numbers | 19.2 | 11.8 | 168.0 | 2.67 | .008 |
| 6a | Number line with guidelines | 16.7 | 14.3 | 131.0 | 0.92 | .360 |
| 6b | Number line without guidelines | 16.3 | 11.1 | 125.0 | 1.72 | .086 |
| 7a | Digit span forwards | 17.0 | 11.7 | 134.5 | 1.71 | .097 |
| 7b | Digit span backwards | 18.9 | 10.9 | 163.0 | 2.54 | .011 |
| 8 words | Oral number comparison | 18.6 | 12.4 | 159.0 | 2.00 | .045 |
| 9 | Non-symbolic quantity estimation | 20.1 | 10.9 | 181.0 | 3.21 | **.001** |
| 10 | Contextual quantity estimation | 17.0 | 14.0 | 135.5 | 1.29 | .198 |
| 11 | Story problem | 19.0 | 12.0 | 164.5 | 2.31 | .021 |
| 12 | Symbolic number comparison | 17.9 | 13.1 | 148.0 | 1.59 | .119 |

Note: Mann-Whitney U test statistic (*U*), *z* = standardized test statistic, *p* = two sided p-values, p-values in bold survived Bonferroni corrected threshold (p = .0031).

1. **Symbolic number comparison – Post-hoc analysis**

As outlined in the introduction, deficits in symbolic number comparison are one of the key impairments observed in children with DD. However, in our study we did not find significant group differences in this ability. Compared to the common symbolic comparison tasks used in literature, the task of our study was a multi-digit symbolic number comparison task that contained up to five digits. We therefore hypothesized that the absence of group difference was in particular related to the performance in TD children in a way, that for younger TD children this task may be more difficult compared to older TD children. We therefore tested whether the TD children that did not reached 100% accuracy were significantly younger compared to the ones that reached 100%. Using independent t test analysis revealed, that the TD children that did not fully succeed in the symbolic number comparison (M = 9.0, SD = 0.7) were significantly younger, *t*(13) = 2.774, *p* = .016, compared to the children that were able to fully succeed (M = 10.1, SD = 0.8) .

1. **Whole brain analyses**

We conducted a whole brain analysis, examining the mean activation across all participants of the contrast number order > number identification by applying a one sample t test, using uncorrected voxel thresholds p < .001 and a minimal cluster size of 10 voxels. Results are displayed in Supplementary Fig. 1 and Supplementary Table 3.


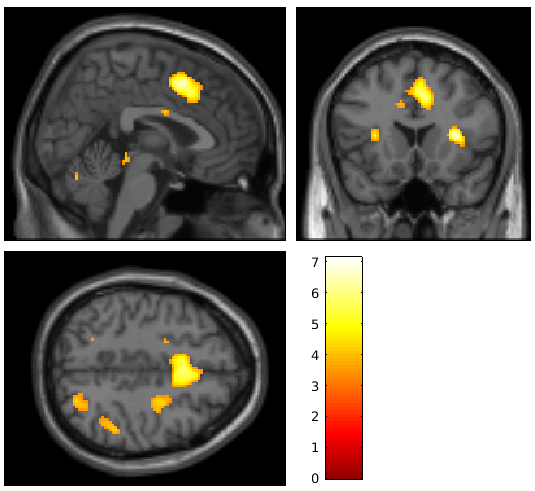


**Supplementary Fig. 1**. Visualization of whole brain analysis (one sample t test) results of the mean activation on the number order vs. number identification contrast across all participants. Minimum T-statistics were set to an uncorrected p = .001 and a minimal cluster size of 10 voxels.

**Supplementary Table 3**: Results of whole brain analyses of mean activation across all participants in the number order vs. number identification contrast.

| Cluster Size | Peak-level | Peak | Peak MNI Coordinates (mm) | | |  |
| --- | --- | --- | --- | --- | --- | --- |
| (Voxels) | *p* (uncorr) | *T* | X | Y | Z | Region |
| 232 | .000 | 7.13 | **32** | **16** | **10** | Right dorsal anterior insula |
| 1436 | .000 | 6.72 | **6** | **10** | **50** | Right pre supplementary motor area |
|  | .000 | 6.02 | **8** | **18** | **40** | Right mid-cingulate cortex |
|  | .000 | 5.43 | **26** | **2** | **54** | Right dorsal premotor cortex |
| 46 | .000 | 5.37 | 0 | -32 | -10 | Unknown area |
| 583 | .000 | 4.92 | **36** | **-46** | **40** | Right anterior intraparietal sulcus hIP1/hIP3 |
|  | .000 | 4.76 | **26** | **-70** | **44** | Right posterior intraparietal sulcus |
|  | .000 | 4.23 | 24 | -64 | 38 | Right posterior intraparietal sulcus |
| 35 | .000 | 4.81 | **2** | **-70** | **-22** | Vermis (lobule VI) |
| 87 | .000 | 4.77 | **-30** | **16** | **12** | Left insula |
| 19 | .000 | 4.53 | **32** | **-62** | **-30** | Right cerebellum (VI) |
| 93 | .000 | 4.38 | **44** | **2** | **32** | Right ventral premotor cortex |
| 26 | .000 | 4.36 | **-32** | **-66** | **-26** | Left cerebellum (VIIa crus / VI) |
| 17 | .000 | 4.36 | 6 | -2 | 28 | Right cingulum |
| 24 | .000 | 4.00 | 16 | -6 | 28 | N. caudate |
| 12 | .000 | 3.98 | 10 | -6 | 4 | Right thalamus (prefrontal) |
| 12 | .000 | 3.85 | -22 | -58 | 44 | Left superior parietal hIP3 |
| 43 | .000 | 3.85 | **-22** | **-66** | **34** | **Left posterior intraparietal sulcus** |
| 22 | .000 | 3.74 | -20 | -2 | 46 | Unknown area |
|  | .001 | 3.49 | -20 | -6 | 54 | Left dorsal premotor cortex |
| 41 | .000 | 3.73 | **-40** | **-4** | **34** | Left ventral premotor cortex |

Note: Peak MNI coordinates in bold were selected initially as the 13 regions of interests.

1. **ROI selection**

The first step in our task-based effective connectivity analysis was the definition of regions that showed a task-related experimental effect. These regions were identified using a whole-brain GLM contrast (Number Order > Number Identification), which revealed 21 peak activations (see Section 4). Second, from these 21 peak activations we selected a group of anatomically and theoretically relevant regions, identified through spatial clustering and prior literature. In this step, we prioritized regions previously implicated in number processing, even when their cluster size was slightly smaller than that of less well-established areas. This resulted in an initial set of 13 ROIs including in the right hemisphere: anterior intraparietal sulcus (aIPS), midcingulate cortex, pre-supplementary motor area (preSMA), and dorsal premotor cortex (dPMC); bilaterally: posterior IPS, anterior insula, ventral premotor cortex (vPMC), and cerebellum; and vermis (see Supplementary Figure 1 and Supplementary Table 3). Third, we assessed whether each participant showed activation within these 13 candidate ROIs. Fourth, based on this information, we aimed to identify the optimal combination of ROIs that would not exceed eight regions, to avoid model overfitting and ensure model stability, while also maximizing the number of participants who could be included in the group-level DCM analysis. This selection was further guided by the theoretical relevance of each region. The final selection included six ROIs: the anterior intraparietal sulcus (aIPS), pre-supplementary motor area (preSMA), dorsal and ventral premotor cortex (dPMC, vPMD), the dorsal portion of the anterior insula (d-aINS) - all in the right hemisphere - and the vermis lobule VI (VER-VI). Participants who did not show activation in all of these six regions (n = 9) were excluded from the group-level DCM analysis.

1. **DCM model comparisons**

To determine the optimal model specification for investigating group differences, we conducted a systematic comparison of Parametric Empirical Bayes (PEB) models with varying combinations of nuisance covariates. While theoretical considerations suggest that demographic variables such as gender, age, and handedness should ideally be included to control for potential confounding effects on the primary covariate of interest (group), empirical model selection based on fit statistics provides a data-driven approach to determine the most parsimonious model.

Six nested models were evaluated:

(a) Group (covariate of interest) only, (b) Group + gender (nuisance covariate), (c) Group + age (nuisance covariate), (d) Group + handedness (nuisance covariate), (e) Group + gender + age (nuisance covariates), and (f) Group + gender + age + handedness (nuisance covariates)

Model selection was performed using the free energy approximation of model evidence, where higher values indicate superior model fit while penalizing for model complexity.

Results:

Model (a) - Group only: F = -1.7973e+05

Model (b) - Group + gender: F = -1.7980e+05

Model (c) - Group + age: F = -1.7980e+05

Model (d) - Group + handedness: F = -1.7977e+05

Model (e) - Group + gender + age: F = -1.7987e+05

Model (f) - Group + gender + age + handedness: F = -1.7992e+05

The results revealed that the simplest model, containing only the group covariate, achieved the highest free energy, indicating the best model fit among all tested specifications.

Models including individual nuisance covariates (models b–d; gender, age, or handedness) each failed to improve model evidence over the group-only model (a). Also models that included combinations of nuisance variables to test whether the nuisance covariates would jointly explain more variance (models e and f) did not account for additional variance. On the contrary, all alternative models with added nuisance covariates showed substantial reductions in model evidence (ΔF > 40), with the full model (f) exhibiting the largest drop (ΔF = 190).

This consistent pattern supports the conclusion that these demographic variables do not substantially confound the group effect in our sample.

1. **Effective connectivity analyses**

All effective connectivity results are displayed in **Supplementary Fig. 2.** with parameters that were different from zero including results with a posterior probability below our threshold of > .99.


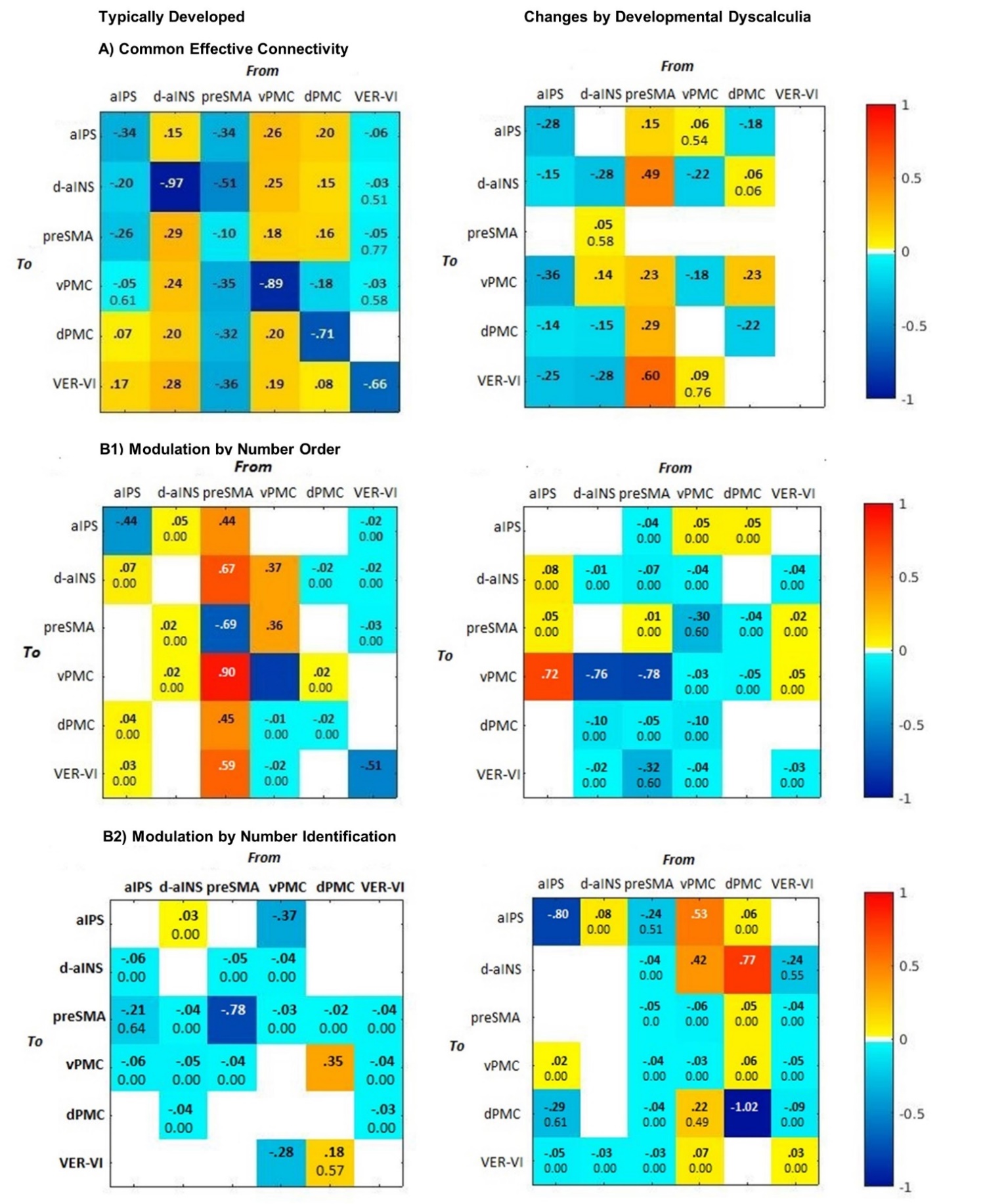


**Supplementary Fig. 2.** Dynamic causal modelling (DCM) results. Results from Bayesian model comparison of parametric empirical Bayesian analyses for typically developed children (TD) on the left and group effect by children with developmental dyscalculia (DD) on the right. The first row refers to A) the common effective connectivity across task conditions (A matrix). The figures in the second and third row represents the B-parameters of B1) the modulation by number order condition and B2) the modulation by number identification which reflect the parameters that additively modulated the common effective connectivity. Parameters with posterior probability of being different from zero of below 99% are labeled with the corresponding parameter value and are color-coded based on the direction of effect. Parameters appearing in white were not different from zero and therefore not explaining the data.
